# Governing digital crisis responses: platform standards and the dilemma of COVID-19 contact tracing

**DOI:** 10.1007/s11573-022-01118-4

**Published:** 2022-11-17

**Authors:** Felix B. Buesching, Dennis M. Steininger, Daniel J. Veit

**Affiliations:** 1 University of Augsburg, Universitaetsstrasse 16, 86159 Augsburg, Germany; 2 University of Kaiserslautern, Kurt-Schumacher-Strasse 74a, 67663 Kaiserslautern, Germany

**Keywords:** COVID-19, Contact tracing apps, Crisis response, Platform governance, Standards wars, Public–private partnership, O3, O33

## Abstract

In response to the impact of the SARS-CoV-2 (COVID-19) pandemic, various developers turned to smartphone-based contact tracing to address the challenges of manual tracing. Due to the presence of network effects, i.e., the effectiveness of contact tracing applications increases with the number of users, information technology standards were critical to the technology’s success. The standardization efforts in Europe led to a variety of trade-offs concerning the choice of an appropriate technological architecture due to the contradictory tensions resulting from the dualism between the need for contact tracing data to contain the pandemic and the need for data minimization to preserve user privacy. Drawing predominantly on the software platform and standards literature, we conduct an interpretive case study to examine the emergence and consequences of this multi-layered decision situation. Our findings reveal how Google and Apple were able to limit the individual leeway of external developers, thereby effectively resolving the European standards war. Furthermore, we identify and discuss the various short-term and long-term trade-offs associated with the standardization of contact tracing applications and translate our findings into recommendations for policy makers with respect to future crisis situations. Specifically, we propose a strategy grounded in our data that enables responsible actors to make goal-oriented and rapid decisions under time constraints.

## Introduction

Since 2019 the coronavirus SARS-CoV-2 (COVID-19) disease began to spread rapidly worldwide. In order to slow down the spread, many governments decided to limit the public and even private social life of their citizens. In addition to social distancing, quarantines, and strict hygiene regulations, several countries opted for digital contact tracing (DCT) through smartphone applications (Altmann et al. 2020). We define applications (hereafter referred to as apps or complements) as “executable pieces of software that are offered as applications, services or systems to end-users” (Ghazawneh and Henfridsson 2013, p. 175). Once users are in close proximity to each other, contact tracing apps record the encounter based on digital technology allowing contacts of positive COVID-19 cases to be quickly notified (Ferretti et al. 2020). DCT apps depend on both, *software platforms* to enable their development and diffusion and *IT standards* to ensure interoperability between different apps and operating systems. The latter stems from the fact that the effectiveness of smartphone-based contact tracing, namely the capability to interrupt infection chains, increases with the number of consumers using the same or an interoperable app (Hinch et al. 2020; Trang et al. 2020). Hence, as opposed to the principle of diminishing returns found in conventional economics (Arthur 1990), DCT apps as well as many other software products exhibit increasing returns to adoption (Arthur 1989), i.e., the value of the technology to a user increases with the number of additional users (Liebowitz and Margolis 1995a), commonly referred to as network effects (Katz and Shapiro 1994).

Whenever network effects are present, *IT standards* are considered to play a fundamental role (Gandal 1995; Weitzel and König 2006). IT standards can be defined as “sets of specifications for communicating or performing actions that ensure that various technologies or products that implement certain specifications are compatible” (Uotila et al. 2017, p. 1208). Thus, standards determine the properties that a technology or product needs to exhibit to ensure interoperability with complementary products (Tassey 2000; Stango 2004). Interoperability in the case of DCT is achieved when different apps can work together and exchange data bi-directionally (Wegner 1996), regardless of their provider or underlying operating system (Zhang et al. 2006). While network effects produce incentives for providers of competing technologies to converge toward a particular standard (Farrell and Saloner 1985), market-based standardization tends to result in battles between various incompatible technologies, commonly known as standards wars (Cusumano et al. 1992; Shapiro and Varian 1999; Stango 2004). Once a particular technology achieves an initial advantage in terms of adoption, it tends to become the dominant standard due to the path dependency resulting from network effects (Arthur 1989), while alternative technologies may be locked out of the market (Schilling 1998). However, under incomplete information, the chosen standard may prove inferior to another in hindsight (David 1985; Liebowitz and Margolis 1995b), yet adopters of the prevailing standard may fail to coordinate their transition to the superior standard (Farrell and Saloner 1985). Such coordination problems result from the fact that “network effects make interdependent decisions of agents that could otherwise be autonomous” (Weitzel and König 2006, p. 491). Large firms may be able to facilitate coordination by driving other actors to the preferred standard based on their sheer size (Farrell and Klemperer 2007). On the other hand, during the development of standards, influential firms may prematurely lock others into an inferior technology and potentially contribute to an overly narrow technological search (Uotila et al. 2017). Hence, in absence of legal obligations to adopt a particular standard (Backhouse et al. 2006), both the development and the diffusion of standards are considered “failure-prone processes” (Markus et al. 2006, p. 440; Gao 2007).

Standards and *software platforms* are strongly intertwined (Hein et al. 2019; Tessmann and Elbert 2022), especially given that network effects constitute a crucial element in driving the overall success of software platforms (Parker et al. 2016; Song et al. 2018). Software platforms such as Google’s Android or Apple’s iOS provide third-party developers (i.e., complementors) access to platform resources to create complementary apps based on the standards defined by the platform (Tiwana et al. 2010; Cusumano et al. 2019). Such platforms mediate two sides of the platform, namely the user and the complementor side, and thus release indirect network effects (Boudreau and Hagiu 2009; Song et al. 2018). Indirect network effects refer to the fact that the value of the platform to complementors increases with the number of users and vice versa (Katz and Shapiro 1994; de Reuver et al. 2018). Therefore, platform owners seek to encourage complementary contributions from third-party developers (Ceccagnoli and Huang 2012). However, they simultaneously need to retain control over the platform (Eisenmann et al. 2006; Ghazawneh and Henfridsson 2013), thus facing a paradoxical tension between generativity and control (Tilson et al. 2010; Tiwana et al. 2010; de Reuver et al. 2018). We follow Li and Kettinger (2021, p. 17) and define generativity as “the software platform owner’s ability to put in place the platform capacity to produce changes mainly driven by external complementors without the direct input from the platform owner” (Tilson et al. 2010; Yoo et al. 2010). To balance such tensions, platform owners leverage boundary resources (e.g., APIs, app stores) that enable and simultaneously govern the value co-creation on software platforms at arm’s length (Ghazawneh and Henfridsson 2013), thereby resolving the aforementioned paradox and aligning the goals of individual platform members with their own (Tiwana 2015; Karhu et al. 2018). Complementors, in turn, rely on boundary resources to access standardized platform resources (Bender 2020) and thus depend on the governance measures of the platform owner (Nambisan and Baron 2013; Hurni et al. 2022). Hence, entrepreneurially activities on software platforms are tied to the prevailing platform rules specified by the platform owner (Huber et al. 2017). However, complementors simultaneously need to act self-determined to satisfy the needs of their customers (Wareham et al. 2014). Considering the presence of both cooperative and competitive dynamics on software platforms (Kang 2017; Wen and Zhu 2019), tensions can arise due to competing values between platform owners and complementors (Selander et al. 2010). Such value competitions are particularly prone to emerge when new technologies are introduced, causing the platform rules and values (Huber et al. 2017), as well as the relationships between the platform members (Selander et al. 2010), to change and dynamically evolve over time (Eaton et al. 2015). Hence, the existing literature shows that both software platforms and IT standards are accompanied by multi-layered tensions and trade-offs (Wareham et al. 2014; Lindgren et al. 2021).

With the onset of the COVID-19 pandemic, several complementors, as well as the platform owners Google and Apple themselves, developed technological protocols that can be used by smartphone apps to perform DCT. The tendency for tensions inherent in IT standards (Lindgren et al. 2021) and software platforms (Mini and Widjaja 2019) manifested in the fact that the protocol preferred by several private and publicly mandated developers focused on the potential epidemiological benefits of DCT (Hern 2020; Newton 2020a; Meyer 2021) while the Google-Apple Exposure Notification (GAEN) protocol jointly developed by both platform owners centered on the preservation of users‘ privacy (Google LLC 2020a; b). While governments, platform owners, and companies alike sought to protect citizens, users, and employees through DCT, initially no consensus could be reached regarding which protocol should be chosen. However, considering the presence of network effects (Hinch et al. 2020), it was of utmost importance to come to an agreement. The question is, however, why exactly was it so difficult to define specifications for DCT protocols that satisfy the interests of all stakeholders? In contrast, while we did observe fierce battles between individual actors initially (Criddle and Kelion 2020), a surprisingly quick agreement was reached due to the broad acceptance of the GAEN protocol across Europe, while alternative technologies and private developers were left behind (Arthur 1989; Schilling 1998). Considering that it is often difficult to determine the ‘best’ technology, even in hindsight (e.g., David 1985; Liebowitz and Margolis 1990), and that Apple and Google according to their own statements did not force anyone to adopt their technology (Etherington and Lomas 2020), the question arises as to how such a quick agreement was achieved? Moreover, already prior to the release of the platform owners’ protocol, alternative protocols from complementors existed, one of which closely resembled Google’s and Apple’s solution. So why was it necessary for Google and Apple to offer their own solution? What are the potential benefits for them? Finally, we find ourselves in the situation that most European governments indeed use the protocol provided by Google and Apple, from which, however, private complementors not affiliated with health authorities are excluded. Whereas the adoption of the platform owners’ proprietary protocol may have been a reasonable decision for governments at the time, the long-term implications are yet to be seen.

While the standardization of DCT (e.g., Marhold and Fell 2021) and especially the involvement of Google and Apple naturally attracted the attention of researchers from various disciplines (e.g., Michael and Abbas 2020; Sharon 2020; Storeng and de Bengy 2021; Lanzing et al. 2022), an extensive investigation based on both the standards and the platform literature addressing the above-mentioned questions and issues remains absent. However, we argue that because DCT apps rely on both *software platforms* and *IT standards* to enable their effective development and the exploitation of network effects, the case provides an avenue to advance the recent research stream that combines the aforementioned bodies of literature (e.g., Hein et al. 2019; Tessmann and Elbert 2022). In particular, the time constraints introduced by the pandemic resulted in trade-offs between short-term and long-term consequences, which, to the best of our knowledge, have not been the focus of research on standards decisions so far (Shin et al. 2015; Lindgren et al. 2021). As critical information is commonly lacking during the technology selection process itself (David 1987), we aim to inform policy makers about the hidden implications of the decisions taken during the COVID-19 pandemic, as well as to provide them guidance for similar situations in the future. Thus, we ask the following research questions:

**RQ1**: How did the complex decision situation surrounding the standardization of DCT unfold?

**RQ2**: How did the platform owners impose their protocol as the technological standard for DCT, and what benefits might this strategy entail for Google and Apple?

**RQ3**: What are the consequences and trade-offs of Google and Apple winning the standards war against complementors?

In order to answer our research questions, we perform an interpretive case study (Strauss and Corbin 1990; Walsham 1995a) by conducting interviews with managers of a consortium behind a privately developed DCT app that was directly affected by Google’s and Apple’s actions. To triangulate our data, we additionally incorporate two extensive interviews with senior staff from the German political arena as well as an informal conversation with the platform owner Google.

## Theoretical foundations

### IT standards

IT standards serve as a critical ingredient in driving the success of innovations by establishing a shared understanding or common “language” of the underlying technology by determining the ground rules (Hanseth et al. 1996; Shin et al. 2015). However, they are commonly considered a “double-edged sword” (Hanseth and Bygstad 2015, p. 646). In part, this ambiguity surrounding the value of standards is reflected in the fact that they need to be stable enough to enable collective actions (Markus et al. 2006). On the other hand, they need to be sufficiently flexible to allow them to be changed when necessary and to be tailored to a wide range of tasks as well as local conditions (Hanseth et al. 1996; Braa et al. 2007). Further, as standards represent interface protocols that can be proprietary resources, they theoretically allow its owner (i.e., sponsor) to lock out competitors to gain a competitive advantage (Lyytinen and King 2006; Zhu and Gurbaxani 2006; Gallagher 2007), thereby potentially raising antitrust concerns (Anton and Yao 1995; Shin et al. 2015). Since predetermined actions and processes are inherent in standards, they can be leveraged as powerful mechanisms to influence other actors in their activities (Hanseth and Monteiro 1997; Backhouse et al. 2006). Dominant (i.e., large) organizations enjoy an advantage in this respect, as their installed user base is less affected by other organizations’ decisions on compatibility with the dominant organization (Farrell and Saloner 1985; Uotila et al. 2017). IT standards can, however, also be unsponsored (Zhu and Gurbaxani 2006). In this case, no actor with a property interest in the standard exists (David and Greenstein 1990; Weitzel and König 2006), and thus its use is not restricted (Stango 2004). A dominant design, on the other hand, can be broadly defined as “a single architecture that achieves dominance in a product category” (Abernathy and Utterback 1978). Hence, dominant designs depend on the market acceptance of an architecture and are rarely controlled by a particular organization (Srinivasan et al. 2006; Gallagher 2007).

While, as shown above, IT standards drive the success of innovations (Shin et al. 2015), technology standardization represents a “failure-prone process” (e.g., Gao 2007) due to the latent tension between the effective development and the diffusion of standards (Markus et al. 2006, p. 440; Lindgren et al. 2021). This tension manifests itself in the fact that even a technologically superior standard can only prevail over alternative technologies if the necessary requirements for its implementation and conditions for its adoption are in place (Lindgren et al. 2021). In this vein, so-called de jure standards often enjoy an advantage, as they are typically mandated by governmental bodies (Shin et al. 2015) and are thus enforced by legal authorities (Lee and Oh 2006; Zhao et al. 2011). They can, however, also emerge without any legal obligation through the consensus of voluntary standards organizations (David and Greenstein 1990). As opposed to de jure standards, de facto standards result from market-based competition between competing technologies (Farrell and Saloner 1988; Stango 2004). Examples of proprietary de facto standards include Google’s Android as well as Apple’s iOS. Such competition can lead to fierce battles between incompatible technologies for market share (Farrell 1996), commonly known as standards wars (Cusumano et al. 1992; Shapiro and Varian 1999). When dealing with unsponsored standards, the new standard is essentially chosen based on demand-side decisions alone, while in the case of sponsored standards, owners can strategically influence users’ behavior (Stango 2004). The main challenges for standards sponsors are achieving legitimacy, ensuring diffusion of the standard, and avoiding its fragmentation through the right level of control without inhibiting the emergence of the standard (Garud et al. 2002). Sponsors with market control can accelerate the competitive process associated with de facto standardization, especially in the presence of network effects (Tassey 2000). Such increasing returns to adoption imply that once a technology gets ahead of the competition and thus enjoys a larger installed base of users, it ultimately tends to become the de facto standard, while other technologies are excluded and may be left behind (Arthur 1989). Sponsors of locked-out technologies may face the problem of not being able to serve the corresponding market unless they adopt the winning standard (Schilling 1998). Some authors argue that this path dependency caused by network effects may lock future users into an inefficient technology and thus into an inferior standard (e.g., David 1985; Cowan 1990). In this vein, Farrell and Saloner (1985) established the term “excess inertia”, i.e., that users with incomplete information may fail to switch to a preferable standard (e.g., Zhu and Gurbaxani 2006), thereby remaining in the (inferior) status quo. Consider, for instance, the QWERTY keyboard, which to this day represents the de facto standard for keyboard layouts, despite the availability of the supposedly superior Dvorak system. Based on this case, David (1985, p. 336) argues that users were prematurely locked into the “wrong system” as a result of path dependency due to increasing returns to adoption. Large actors may reduce the risk of such coordination problems by incentivizing smaller actors to follow them in adopting the superior standard (e.g., Farrell and Klemperer 2007). Uotila et al. (2017), on the other hand, find in their study on standards development that coordination through influential actors can lead to an overly narrow search, leaving the ‘best’ standard undiscovered. They further show that if such influential actors are not powerful enough, the risk arises that they may, under imperfect coordination, lead other actors into a premature lock-in by creating a bandwagon effect (e.g., Wade 1995) which they will then be unable to override. Thus, while empowering large actors for the sake of resolving coordination problems can be beneficial, such situations can at the same time introduce pitfalls such as lock-in (and lock-out) effects as well as an overly narrow technological search during the standard development process, which might conflict with the public interests of an industry as a whole (Lindgren et al. 2021). Others, like Liebowitz and Margolis (1995a, b), challenge the notion of lock-ins and market failures in this regard altogether. Staying with the previously mentioned example of keyboard layouts, the authors question the superiority of the Dvorak layout and argue that the QWERTY keyboard constitutes a reasonably viable technology (Liebowitz and Margolis 1990). Drawing on other alleged lock-in scenarios, the authors conclude that “good products win” and that “people choose what they want” (Liebowitz and Margolis 1999, p. 235). While they do not dismiss the fact that poor decisions occur, they argue that such inefficiencies are resolved on their own as they lead to profit opportunities that are eventually exploited. In summary, the analysis of the existing literature demonstrates that especially the market-based standardization of incompatible technologies is a complex undertaking, and the question of the ‘best’ technology is not trivial to answer, even in retrospect (e.g., David 1985; Liebowitz and Margolis 1990). In summary, as shown in Table 1 and explained above, three latent tensions arise from technological standardization, namely the tension between stabilization and flexibility as well as between development and diffusion activities, in addition to the recently mentioned tension between public and private interests (Lindgren et al. 2021).

**Table 1:** Tensions in IT standardization. Adapted from Lindgren et al. (2021)

Tension	Development vs. diffusion	Private vs. public interests	Stability vs. flexibility
Example	Standardization activities are considered a failure-prone endeavor, bound to both the effective development of standards and the creation of adequate conditions for their adoption	Empowering large actors can be beneficial in addressing coordination problems, but can simultaneously introduce pitfalls, such as an overly narrow technological search, as well as lock-in/lock-out effects	Standards must be stable enough to ensure compatibility through a common understanding of the technology, while at the same time they need to be flexible enough allowing them to be changed and adapted to their area of application
Literature	Markus et al. (2006)	Uotila et al. (2017)	Hanseth et al. (1996)

### Software platform ecosystems

Software platforms can be defined as “the extensible codebase of a software-based system that provides core functionality shared by the modules that interoperate with it and the interfaces through which they interoperate” (Tiwana et al. 2010, p. 675). Under this definition, the core architecture of platforms consists of technological building blocks or modules that can be shared by the platform owner with third-party developers (Cusumano et al. 2019), commonly referred to as complementors. By exposing application programming interfaces (APIs), the platform modules are made accessible to the complementors, who then combine those modules to build and innovate complementary apps and services based on the standards set by the platform (Eaton et al. 2015; Wulf and Blohm 2020; Bonina et al. 2021). The reprogrammability of digital technology allows the platform features to be extended without explicitly having been intended by the system originator (Yoo et al. 2010; Lyytinen et al. 2016). Hence, software platforms can also be referred to as innovation platforms (e.g., Cusumano et al. 2019; Gawer 2020; Bonina et al. 2021). As exemplified by Google and Apple, owners can link software platforms to dedicated transaction platforms (e.g., app stores), serving as intermediaries between users and developers (Karhu et al. 2020). By bringing users together with complementors, software platforms release indirect network effects, meaning that both sides of the platform influence each other’s growth (Katz and Shapiro 1985; Evans 2003; Song et al. 2018). For the present study, we adopt an ecosystem perspective, consisting of the platform owners, complementors, and users, as well as the governance mechanisms that enable the co-creation of value (Hein et al. 2020). Platform governance essentially translates into “who makes what decisions about a platform” (Tiwana et al. 2010, p. 679). A key activity to govern software platforms is control, allowing the strategies and goals of complementors to be aligned with those of the platform owner (Tiwana 2015). The exercise of control by the platform owner is required since complementors are driven by self-interested motives and therefore act entrepreneurially to meet the needs of their customers (Wareham et al. 2014; Hurni et al. 2022). Hence, platform owners and complementors typically do not represent a classical firm-supplier relationship (Jacobides et al. 2018).

#### The boundary resource model

Platform owners need to stimulate and facilitate the generative efforts of complementors in a way that allows them to contribute new and innovative complements to the platform (Ghazawneh and Henfridsson 2013). The co-creation of value between platform owners and third-party developers on software platforms, therefore, causes a paradoxical tension between generativity and control (Tilson et al. 2010; Tiwana et al. 2010; de Reuver et al. 2018), as platform owners seek to release indirect network effects by stimulating complementary contributions from external developers while simultaneously pursuing control over the platform (Eisenmann et al. 2006; Ghazawneh and Henfridsson 2013). Existing literature suggests that boundary resources, serving as the digital interface between the platform and its complements (Gawer 2020), resolve the generativity-control paradox (e.g., Ghazawneh and Henfridsson 2013; Eaton et al. 2015; Karhu et al. 2018). Following Ghazawneh and Henfridsson (2013, p. 175), we define boundary resources as “the software tools and regulations that serve as the interface for the arm’s length relationship between the platform owner and the application developer.” Boundary resources on software platforms include, for instance, APIs (Wulf and Blohm 2020), software libraries (Fink et al. 2020), app stores (Karhu et al. 2018), and software development kits (SDKs) (Gawer 2020). Platform owners design boundary resources to capitalize on contributions (resourcing) by complementors and to ensure control (securing) over the platform (Ghazawneh and Henfridsson 2013; Karhu et al. 2018). Although platform owners design boundary resources, they evolve and are dynamically tuned over time through the collaborative activities of various stakeholders (Eaton et al. 2015). When boundary resources are perceived as insufficient, the platform owner typically seeks to either adapt existing ones or introduce new ones, often accompanied by modified rules to ensure the owner’s control (Ghazawneh and Henfridsson 2010). Consider, for example, Apple, which initially refused to open up its platform to native apps from third-party complementors, but later provided the necessary SDKs and the Apple App Store under pressure from complementors and users, while at the same time taking steps to block apps from sources other than Apple (Yoo et al. 2012; Eaton et al. 2015).

#### Control in software platform ecosystems

Boundary resources can further be synthesized with the control literature (Ouchi 1977; Kirsch 1997) to illustrate the kind of control they provide. The integration of marketplaces such as app stores enables complementors to distribute and monetize their complements (Karhu et al. 2018) while allowing the platform owner to act as a gatekeeper (Zhang et al. 2020). Such input control can be defined as “the degree to which the platform owner uses predefined objective acceptance criteria for judging what apps and app developers are allowed into a platform’s ecosystem” (Tiwana 2014, p. 124). The fact that input control measures allow platform owners to determine who or what is allowed into the ecosystem naturally implies a concomitant right to exclude external developers and their respective complements from the platform referred to as the “bouncer’s right” (Strahilevitz 2006; Boudreau and Hagiu 2009; Tiwana 2014). Consequently, an exclusion via input control poses a prevailing threat to developers due to the dependency on app stores (Qiu et al. 2017). Boundary resources can further enable the exercise of process control. Consider, for example, SDKs. While they typically represent an act of resourcing by facilitating app development (Gawer 2020), they simultaneously enable securing the platform (Goldbach et al. 2018), as platform owners use them to specify the scope in which third-party developers can create complementary services and products (de Reuver et al. 2018; Goldbach et al. 2018). Process control, also referred to as behavior control (e.g., Mukhopadhyay et al. 2016; Mukhopadhyay and Bouwman 2018), involves predefined procedures and methodologies to which the controlled party must adhere (Goldbach et al. 2014, 2018). The adherence of the complementors to the prescribed processes and guidelines ultimately achieves outcomes desired by the platform owner (Kirsch 1997) without having specified them in advance (Goldbach et al. 2014). Note that these outcomes desired by the platform owner do not indicate the competitiveness of complements but rather the interoperability with the platform (Tiwana 2014).

Another way for platform owners to increase control over the entire ecosystem is to integrate the functionality of a third-party complement directly into the platform core (Bender and Gronau 2017), a process known as “coring” (Bender and Gronau 2017; Bender et al. 2019). In the case of smartphones, coring is exemplified by updates to the operating system that add enhanced features implemented by the platform owner (Bender and Gronau 2017). Coring poses a risk to complementors in terms of monetization, as they depend on boundary resources in order to innovate (Kang 2017; Bender et al. 2019; Bender 2020). However, this, in turn, increases the potential for the functions of individual complements to be integrated into the platform core (e.g., operating system) by the platform owner (Bender et al. 2019; Bender 2020), thereby making the complement obsolete. Hein et al. (2019) observe in a similar vein that platform owners aggregate complements with overly specific use cases and provide them to the entire ecosystem as novel boundary resources. While this kind of substitution of complements presents a risk for individual complementors, other complementors within the ecosystem generally benefit from the extended functional scope of the platform core or the provision of additional boundary resources in their generative activities (Bender and Gronau 2017; Hein et al. 2019).

## Research design

### Study setting

We examine the case predominantly from the perspective of the third-party complementors behind the DCT app called TraceCOV. TraceCOV was developed by a joint initiative of several German companies.^1^ The driving forces in this consortium are the companies TraceCo Germany and CrowdCo. TraceCo Germany is an auditing and consulting firm that refers to an international network consisting of several legally independent companies. One member of this network is the consulting firm TraceCo Consulting, which is also part of the consortium behind TraceCOV. For the sake of readability, we refer from now on only to TraceCo without the addition *Germany* or *Consulting*. Alongside TraceCo, the firm CrowdCo plays a decisive role in the TraceCOV project. The German company is primarily active in the field of digital crowd management and specializes in the analysis of crowd behavior using smartphone data. Although TraceCOV is distributed internationally, this study focuses mainly, but not exclusively, on the European market. The reason for this is that TraceCOV was developed in Germany, and the roll-out started from there. One of the key features of TraceCOV, in addition to contact tracing, is the ability for corporate customers to integrate their internal test management into the app. This integration is primarily intended for companies that test their employees for COVID-19 in periodic cycles. To triangulate our data and to examine the case holistically, we additionally interviewed a high-ranking Google manager and senior staff from the German political arena. The latter interviews involved, on the one hand, an employee of the German Ministry of Health, which was largely in charge of the decisions concerning the official German contact tracing app called Corona-Warn-App. The remaining interviewee was informed about the Corona-Warn-App as an employee of a German parliament member belonging to the Committee on Digital Affairs. Table 2 describes the actors involved in terms of their roles.

**Table 2:** Description of actors

Actor	Google (& Apple)	TraceCo	CrowdCo	Political actors
Role	Owners of the operating systems and associated marketplaces to which the development and distribution of DCT apps are tied	Platform complementors with the objective of providing their DCT app to other organizations, thereby reliant on the ecosystems of Google and Apple	Partner of TraceCOV concerning their DCT app and providing the technological know-how for the development of the app	Public authorities providing DCT apps to their citizens, thus acting as platform complementors. In Germany represented by the Ministry of Health

### Case description

**Fig. 1:**
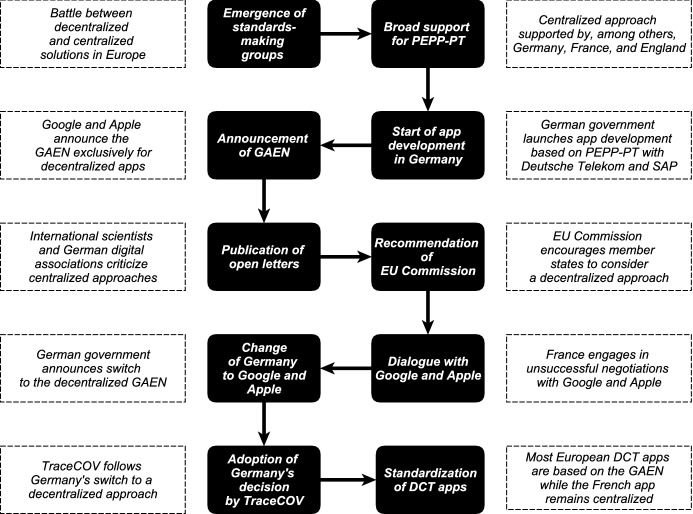
Standardization of digital contact tracing apps: chronology of major events

During the COVID-19 pandemic, two distinct types of DCT have gained widespread recognition: surveillance and proximity tracing. Surveillance tracing is characterized by the use of location data and other digital data such as credit card records or social media data to trace contacts *retrospectively* (Riemer et al. 2020). While surveillance tracing is predominantly seen in Asian countries (Nageshwaran et al. 2021), it has not gained the same momentum in Europe due to its inherent privacy-invasive nature. Most European governments opted for proximity tracing approaches that use digital technology to collect smartphone data *while* contacts occur between users (Riemer et al. 2020). To collect this data, two technologies have become established in the international debate on the appropriate architecture of proximity tracing: GPS and Bluetooth. Besides the privacy concerns arising from the fact that location data needs to be centrally collected, GPS data is not considered accurate and precise enough to perform effective contact tracing (e.g., Merry and Bettinger 2019). Hence, European developers predominantly opted for Bluetooth-based contact tracing apps. Since our study focuses mainly on Europe, we will use the terms DCT and proximity tracing interchangeably. The architecture of Bluetooth-based DCT apps can be differentiated between decentralized (e.g., DP-3T protocol) and centralized (e.g., PEPP-PT protocol) approaches. The main difference between the two approaches is whether the COVID-19 exposure estimation, which needs to exceed a certain threshold to trigger a notification of the user, is carried out locally on the smartphone or a central server (Boutet et al. 2020). While more research is needed to determine which approach might be more effective, initial studies including both centralized and decentralized apps suggest that both approaches can be effective in reducing the spread of COIVD-19 (Urbaczewski and Lee 2020). While existing research indicates that both approaches entail privacy risks (e.g., Vaudenay 2020a; White and van Basshuysen 2021a), the main argument in the European debate was based on decentralized approaches eliminating the risk of data breaches inherent in centralized systems. Nevertheless, some European governments (e.g., Germany, England, France) initially advocated centralized DCT apps due to epidemiological benefits such as the potential of carrying out evaluations and the integration into manual contact tracing (Riemer et al. 2020), thus potentially outperforming decentralized approaches in suppressing virus transmission (Plank et al. 2020; White and van Basshuysen 2021b; Elmokashfi et al. 2021). The debate about the appropriate protocol for DCT apps divided Europe into two camps and led to a market-based standards war between the incompatible technologies (i.e., decentralized and centralized protocols) (Cusumano et al. 1992; Shapiro and Varian 1999), which was ultimately resolved by Google and Apple due to the introduction of the GAEN. Figure 1 provides a summary of the major events in our case study.

### Methodology

Due to the uniqueness of the case and its complex context, we adopt a single case study approach, as it allows an in-depth analysis and a holistic description of the specific case to answer “how” and “why” questions (Walsham 1995a; Yin 2009). To examine the beliefs and experiences of the participants in our interviews, we employ an interpretive stance (Walsham 1995a, b). We place considerable weight on a comprehensive description of the case context and on capturing the opinions and thoughts of the interviewees. Considering that the case under investigation in this study has been very dynamic and unpredictable since the outset of our research, we initially remained open-minded and continuously refined the sampling strategy through an iterative process of joint data collection and analysis (Glaser and Strauss 1967). To address theoretical saturation and to provide a thick description of the case context, we additionally draw on archival data (see Table 4). This approach facilitates the avoidance of biases in interpretive case studies potentially caused by misinterpretations of statements (Ghazawneh and Henfridsson 2013).

As the primary source for the collection of our data, we initially conducted eleven formal interviews (see Table 3) between July 2020 and November 2020 with members of the TraceCOV project. We ensured to cover a wide range of roles to represent the case holistically. The data collected in the interviews were supplemented with marketing materials and information obtained from the official TraceCOV website. We additionally conducted interviews with other stakeholders to triangulate our data and address theoretical saturation. Specifically, we conducted two additional formal interviews with senior staff from the German political arena and one informal interview with a high-ranking Google manager (see Table 3). We use the terms formal and informal to distinguish between the interviews (e.g., Schultze 2000; Chanias et al. 2019), as the former were audio-recorded and transcribed, while notes were taken during the informal interview with Google (PO#1). The interview with Google was primarily intended to gain deeper insights into the emergence and regulations behind the proprietary contact tracing protocol developed by Google and Apple. The interviews with staff from the German political arena focused mainly on the Corona-Warn-App. On average, each interview lasted about 30–40 min. The chosen time frame was based on the availability of the interviewees. Due to the ongoing pandemic at the time of the data collection, we have refrained from field visits. The interviews were conducted via video conferencing in the native language (i.e., German) of the interviewees. Hence, in-text quotes were translated into English. Names were pseudonymized in the transcripts, as shown in Table 3. A distinction is made between the first (.1) and the second (.2) interview for individuals who have been interviewed twice. Follow-up interviews were essential to capture the dynamics of the case. In terms of our sampling strategy, we followed the data of previous interviews to determine what data we would collect next (Glaser and Strauss 1967). We adopted a semi-structured approach for carrying out the interviews (Myers and Newman 2007). A script with key questions was prepared for each interview. We chose to follow the same semi-structured questionnaire for the interviews with the employees from the German political arena allowing us to eliminate potential biases since we were able to capture both the internal perspective of the German Ministry of Health and how their decisions were reported and perceived in the parliament.

**Table 3:** Summary of the interviews conducted

n	Organization	Role	Main theme(s)	Pseudonym	Date
1	TraceCo Consulting	Head of Marketing	Initial interview, GAEN	D#1.1	03.07.2020
2	TraceCo Germany	Project Leader on Operational Level	Project initialization	D#2	09.07.2020
3	CrowdCo	Head of Technological Partner	DCT technology, GAEN	D#3.1	31.07.2020
4	TraceCo Middle East	Project Leader on Operational Level	Customer perspective	D#4	14.08.2020
5	TraceCo Consulting	Project Sponsor	Deployment & marketing	D#5.1	14.08.2020
6	TraceCo Germany	Sales & Key Account Manager	Sales interview	D#6	02.09.2020
7	TraceCo Consulting	Head of Marketing	Roll-out progress	D#1.2	11.09.2020
8	TraceCo Germany	Coordinator Frontend Team	Frontend & deployment	D#7	25.09.2020
9	TraceCo Germany	User	User perspective	D#8	22.10.2020
10	CrowdCo	Head of Technological Partner	DCT technology, GAEN	D#3.2	30.10.2020
11	TraceCo Consulting	Project Sponsor	Follow-up & outlook	D#5.2	06.11.2020
12	Google EMEA	Director Business Development	GAEN	PO#1	16.05.2022
13	German Parliament	Committee on Digital Affairs	Corona-Warn-App, GAEN	G#1	15.06.2022
14	German Ministry of Health	Corona-Warn-App	Corona-Warn-App, GAEN	G#2	17.06.2022

The data collection and analysis were carried out in a concurrent manner (Glaser and Strauss 1967). This overlap proves to be advantageous in that unique or unexpected themes emerging from the data analysis can be pursued flexibly and opportunistically throughout the research process (Eisenhardt 1989). Concerning our data analysis, a three-step coding approach was used (Gioia et al. 2013). We adopted an incident-by-incident approach (Charmaz 2008). First, we investigated what exactly happens in the data and whether concepts and categories can be derived from the data (Glaser 1978). Thus, we were able to label meaningful incidents in our data with descriptive codes that remained close to the original evidence (Strauss and Corbin 1990; Gioia et al. 2013). In the second coding stage, we interpreted the broken-down data while being alert for similarities and differences to reunite our open codes to higher-order themes (Gioia et al. 2013). At this point, it should be noted that we collected publicly available archival data (n = 78) to address theoretical saturation with respect to our research questions (see Table 4). In line with previous qualitative research (e.g., Chanias et al. 2019), our archival records were arranged chronologically within a single document allowing us to code them along with the interview data. We added interviews and archival documents until we were not able to find any new views or additional insights on the phases and tensions of our case.

**Table 4:** Summary of the archival data used (n = 78)

Type	Exemplary sources	References
Public correspondence	Governments; Ministries of Health; European Commission; European Center for Digital Rights	CNIL (2020), Ministry of Health Singapore et al. (2020), Publications Office of the European Union (2020), European Commission (2020, 2021), European Center for Digital Rights (2020), Bundespresseamt (2020a; b, c), Bundesgesundheitsministerium (2020) and Government of Singapore (2020)
Corporate correspondence	Google; Apple	Apple Inc. (2020a, b, c) and Google LLC (2020a; b, c, d, e, f)
White papers and websites of DCT protocols	DP-3T, PEPP-PT; ROBERT; BlueTrace	PEPP-PT (2020), Bay et al. (2020), DP-3T (2020), Castelluccia et al. (2020) and Troncoso et al. (2020)
Scientific documents	Research articles, technical reports, and research reports on DCT apps and protocols	Farrahi et al. (2014), Ahmed et al. (2020), Azad et al. (2020), Vaudenay (2020a), Boutet et al. (2020, 2021), Zastrow (2020), Li et al. (2020), Kleinman and Merkel (2020), Sharon (2020), Rowe et al. (2020), Cebrian (2021), Krehling and Essex (2021), Wang et al. (2021), White and van Basshuysen (2021b) and Schultz et al. (2022)
News articles	Forbes; The Washington Post; The New York Times; Financial Times; Bloomberg; The Guardian; Fortune; BBC News; Reuters	Barbaschow (2020), Baumstieger et al. (2020), Francisco (2020), Hurtz (2020), Kelion (2020a, b, c), Schurter (2020), Horowitz (2020), Burton (2020), Busvine (2020a, b), Lomas (2020a, b), Gold (2020), Chee (2020), Doffman (2020a, b), Fouquet (2020), Hern (2020), Etherington and Lomas (2020), Kelly (2020), Newton (2020a, b), Vincent (2020), Criddle and Kelion (2020), Abboud et al. (2020), Albergotti and Harwell (2020), Scott et al. (2020), Morrow (2020), Dillet (2020), Gladstone (2020), Ilanbey (2021) and Meyer (2021)
Open letters	German data privacy and security associations; international researchers	Kaafar et al. (2020) and D64 et al. (2020)

**Fig. 2:**
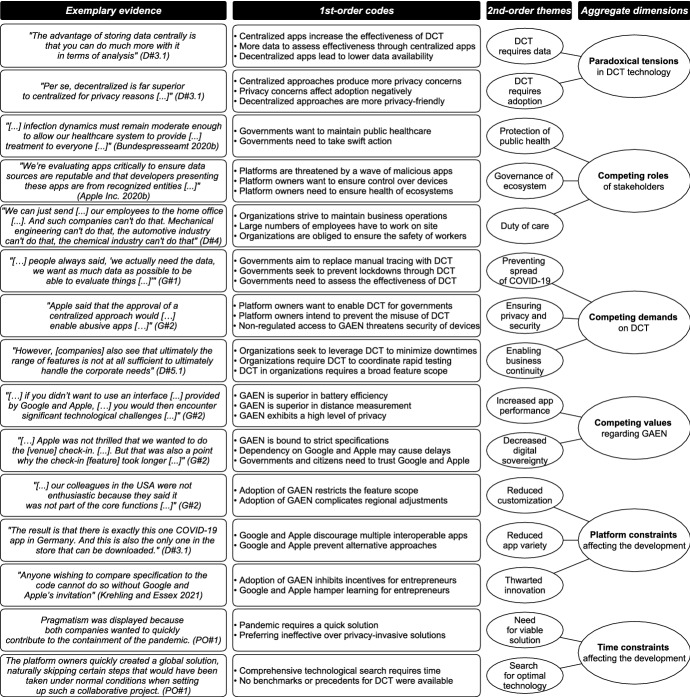
Data structure including exemplary evidence

Due to the continuous stream of new data, we went back and forth in the data and refined the codes accordingly. In a final step, we identified several core categories (Halaweh et al. 2008). In this process, we abstracted the themes identified in the previous step to aggregate dimensions in order to make sense of our data (Gioia et al. 2013). We then moved from the description of the case to the deductive explanation of the core phenomenon (Vaast and Walsham 2013). We, therefore, examined the standards, the platform, and the control literature to explain our data by theoretical concepts (Walsh and Bartunek 2011). Our observations, categories, and codes were constantly compared both between and within the interviews (Strauss and Corbin 1990). This constant comparison ensured both precision and consistency when coding the incidents in our data (Vaast and Walsham 2013) and facilitated the identification of meaningful categories. Furthermore, this technique enabled us to remain suspicious of our data and sensitive to possible distortions in the interpretations of the interview participants (Klein and Myers 1999). Besides the application of constant comparison, the preparation of memos accompanied the entire analysis procedure (Strauss and Corbin 1990). Memos were created via audio recordings to express and capture our thoughts about the data and the entire research process. The software ATLAS.ti was used for the coding procedure. A condensed data structure outlining how we arrived at theoretical abstraction for our overarching model is shown in Fig. 2 along with exemplary evidence (Gioia et al. 2013). Furthermore, the Appendix provides a detailed and comprehensive account of our data analysis.

## Results

In the following sections, we describe the standardization of DCT apps across Europe, focusing primarily on the privately developed app called TraceCOV and the official German app called Corona-Warn-App. We present the results through a thick description of the specific context and situate the case in a chronological narrative.

### Pre standards war: digital technology to assist in the pandemic response

On March 20, 2020, Singapore’s Government Technology Agency and the Ministry of Health released the proximity tracing app called TraceTogether (Ministry of Health Singapore et al. 2020). The app was developed in response to the increasing difficulties encountered using traditional contact tracing (Burton 2020). By developing TraceTogether, which was inspired by Farrahi et al. (2014), Singapore pioneered the field of Bluetooth-based DCT (Cebrian 2021). When using Bluetooth-based DCT apps like TraceTogether, mobile devices use Bluetooth Low Energy (BLE) to exchange identifiers when in close proximity and store them locally in a contact history log (Troncoso et al. 2020). If a user tests positive for COVID-19, their contacts can be traced via the identifiers stored in the history log and thus be notified. Shortly after the launch of TraceTogether, more than 50 governments expressed their interest in the solution, prompting the Singaporean government to release the underlying protocol BlueTrace and its reference implementation OpenTrace as open source (Bay et al. 2020). The centralized BlueTrace protocol was initially met with considerable enthusiasm and was generally considered as worth emulating (Criddle and Kelion 2020). However, it suffered from the limited Bluetooth background functionality due to Apple’s restrictive policy regarding its operating system iOS. Due to Apple’s regulated platform resources, TraceTogether had to run in the foreground on iOS devices (Bay et al. 2020; Kleinman and Merkel 2020). iPhone users had to keep their device unlocked and TraceTogether open, causing inconveniences for users and significant battery drain (Zastrow 2020). Ironically, in addition to protecting users’ privacy, avoiding battery drain was the reason why Apple integrated the policy (i.e., blocking BLE) in the first place (Albergotti and Harwell 2020).

After the release of TraceTogether, TraceCo and CrowdCo quickly recognized that they were capable of developing their own DCT app following the Singaporean model. In joint projects between the two companies before the pandemic, CrowdCo’s crowdsensing technology was already applied to several cases closely related to DCT. The main difference was that those earlier applications of the technology were based on GPS data. However, Bluetooth has emerged as the preferred technology for DCT, as GPS is not considered accurate enough (Merry and Bettinger 2019). Nevertheless, TraceCo and CrowdCo succeeded in adapting an existing solution from previous projects to proximity tracing based on BLE. Therefore, as D#3.1 explained, the solution as such remained viable:

*At the end of the day, the backend does not care whether it is processing location data or contact tracing data. So, the whole backend that manages and efficiently plays out all the infection IDs needed for contact tracing and potentially on millions of devices, that was already there. […] We simply took another app, which we already had as a demo app, as a basis and then built the product based on it. (D#3.1)*

However, the team behind TraceCOV initially encountered limitations concerning the exploitation of iOS platform resources, namely the restricted Bluetooth background functionality known from TraceTogether. Australia’s multimillion-dollar DCT app, which at the time was based on BlueTrace (Tannock and McClymont 2020; Kelly 2020), indicated this limited access to iOS platform resources not only causes inconveniences for iPhone users but may also contribute to the ineffectiveness of DCT apps. In Victoria, a state in the southeast of Australia, the app detected no contacts between April 2020 and the end of July 2020 that were not already identified by traditional contact tracing (Gladstone 2020). To solve this issue, CrowdCo developed a workaround to bypass Apple’s restrictions. The workaround ensures that TraceCOV is indeed capable of running in the background, but only under the condition that iPhones are not isolated from other smartphones for an extended period of time. As soon as two iPhones are in close proximity, both devices keep each other awake and thus prevent contact tracing from stopping in the background. In addition, the team behind CrowdCo developed a mechanism that allows Android devices to keep iPhones active and re-activate them. Thus, in case an iPhone is isolated for a prolonged period, it can be re-activated by Android devices. By bypassing the limitations of iOS, TraceCOV’s developers were operating at the cutting edge of what was technologically feasible at the time, as D#2 stated:

*The national app in Singapore, which was launched relatively early on, could not run in the background at all. […] And then we solved that three weeks later […]. So that should give a sense of how much one is at the front of what is technically possible at this point. (D#2)*

TraceCOV consequently belonged to the pioneers in this field and aimed to position itself as the national DCT app in Germany by getting the development contract from the German government.

### Standards war: battle between incompatible technologies

In March 2020, COVID-19 cases in Europe surged, with Italy and Spain severely affected, prompting European governments to mandate lockdowns (Horowitz 2020). In its crisis response, Europe adopted the Asian model and increasingly relied on digital solutions. Already in April 2020, several European health authorities and private companies were working on DCT apps (European Center for Digital Rights 2020), resulting in a patchwork of solutions (Chee 2020). Recognizing this fragmentation, the European Commission advocated an overarching and interoperable solution among its member states (European Commission 2020) in hopes of easing restrictions and lockdowns (Baumstieger et al. 2020). Whereas many Asian countries have established surveillance tracing mechanisms (Doffman 2020b), such approaches were not conceivable in Europe due to stricter data protection laws such as the General Data Protection Regulation (GDPR) (Lomas 2020a). Therefore, the public debate around DCT in Europe has been characterized by data privacy and security considerations (Sharon 2020).

Driven by the debate about interoperability and privacy, a group of 130 developers and researchers launched the Pan-European Privacy-Preserving Proximity Tracing (PEPP-PT) project (Gold 2020). The group announced that, as its name suggests, it is working on a privacy-preserving European standard to facilitate proximity tracing based on BLE (PEPP-PT 2020). Like the Singaporean BlueTrace protocol, PEPP-PT comprised an open standard that platform complementors can integrate to develop DCT apps. By using country codes, the PEPP-PT protocol aimed to provide interoperability across national borders (Busvine 2020a). The group announced it would refrain from collecting location data and comply with European privacy laws (Abboud et al. 2020). At the time, the initiative was backed by Germany, France, Italy, Spain, Switzerland, Austria, Belgium, Denmark (Hurtz 2020; Schurter 2020), and also by TraceCOV. Germany was expected to be the first governmental authority to integrate the protocol into its app (Doffman 2020a). The German government publicly announced in mid-April 2020 that it would build its app on the PEPP-PT protocol (Bundespresseamt 2020a). Furthermore, it encouraged complementors working independently on DCT solutions to base apps on PEPP-PT in the interest of interoperability. Ultimately, however, the German government did not partner with TraceCo and CrowdCo but opted for both SAP and Deutsche Telekom to develop its official app (Bundespresseamt 2020b). G#2 emphasized that the decision to work with both Deutsche Telekom and SAP was politically motivated, as both companies were already involved in initial research projects surrounding contact tracing led by the Fraunhofer Institute:

*[…] SAP was already involved in advising Fraunhofer when it became clear that the complexity was becoming too great for this research institute, and T-Systems (i.e., Deutsche Telekom) had already been entrusted with hosting issues when it became clear that the small hosting solutions that had been considered up to that point would have been massively overwhelmed by the data traffic caused by such apps. (G#2)*

After the initial goal of becoming the national German DCT app was no longer viable, the TraceCOV consortium reoriented itself to continue utilizing the existing solution. Hence, the strategic decision was made to adjust the app and its target group to corporate customers. Although government-sponsored apps were either already in use or undergoing development in several countries around the world, the consortium did not consider this an issue concerning their solution:

*Our approach was actually from the outset [...] to create a whole portfolio of apps that all have this one component of contact tracing in them so that different segments of the market can be served by different players. The important thing is that they are all compatible with each other. (D#3.1)*

The compatibility that D#3.1 was alluding to would have been achieved by aligning the various apps to a pan-European standard such as PEPP-PT. However, the centralized PEPP-PT solution faced competition from the Decentralized Privacy-Preserving Proximity Tracing (DP-3T) project. The DP-3T protocol was developed by a team of over 25 scientists from various European countries (DP-3T 2020). The main difference between the two approaches is whether the virus exposure estimation is carried out locally on the smartphone (DP-3T) or on a central server (PEPP-PT) (Boutet et al. 2020). Note that a central back-end server is required even in the case of decentralized protocols. Centralized approaches, which were at the time used by TraceTogether and TraceCOV, offer epidemiological advantages, as noted above, because statistical evaluations can be carried out. Therefore, the team behind TraceCOV considered centralized approaches beneficial:

*On a meta-level, it would have been totally exciting from an epidemiological and infection- and pandemic-scientific point of view to do investigations, make analyses, identify hotspots, and so on and so forth. (D#2)*

*The advantage of storing data centrally is that you can do much more with it in terms of analysis. [...]. You have a comprehensive overview of the entire behavior of your population in an anonymous way and also only regarding their contact behavior. (D#3.1)*

The benefits of centralized approaches were also noted in the political arena in Germany:

*[…] there are arguments to follow the centralized approach because then you have a minimum set of data that allows you to evaluate what you are doing. (G#2)*

*[…] people always said, “we actually need the data, we want as much data as possible to be able to evaluate things to combat the corona crisis.” It would have often spoken for the centralized approach. (G#1)*

The analyses mentioned by D#2 and D#3.1 are possible because user data is processed on a central server maintained by a responsible authority (e.g., public health authority) (Zastrow 2020). While the data provide more resources to contain the pandemic (Azad et al. 2020), it theoretically allows relevant authorities to access information about the health status (i.e., infected, exposed, uninfected) of users (Vaudenay 2020a; Li et al. 2020). Decentralized approaches aim to protect users from malicious attackers at the state level. For this reason, the central server is trusted with as little information as possible (Troncoso et al. 2020). Hence, the main argument in the public debate was based on decentralized approaches eliminating the risk of data breaches inherent in centralized protocols. D#3.1, however, emphasized that it is very well possible to design centralized DCT apps in a way that preserves privacy:

*If we say the goal is that the owner of the app wants to drive analytics, the centralized [approach] is much better suited, but it has this big “trust disadvantage,” let’s call it that, because per se, you certainly can also design it in a way that is privacy-preserving. (D#3.1)*

For Germany, too, it was clear from the very beginning that, regardless of the approach, users’ privacy needs to be preserved:

*[...] the anonymity of the approach was never in question. The centralized approach was also a data-efficient approach in terms of its fundamental idea. After all, it was only about hosting anonymized data in the background. (G#2)*

*So, data protection and data security. [...] This is something that you don’t really touch politically. It must be a basic prerequisite, so to speak, that everything is done in compliance with data protection. And I would always say from the parliamentary point of view, “we’d rather the thing doesn’t work so well than have a problem at the data protection level.” (G#1)*

Ultimately, a standards war emerged that divided Europe into two opposing camps (Criddle and Kelion 2020), complicating and prolonging the deployment of DCT apps based on a pan-European standard. While countries like Germany, England, and France remained with those advocating a centralized standard such as PEPP-PT, others like Switzerland and Austria favored the decentralized DP-3T protocol due to privacy concerns (Busvine 2020b).

### End of the standards war: intervention by the platform owners

In addition to the public criticism of centralized approaches, the announcement of the Google-Apple Exposure Notification protocol on April 10, 2020, has significantly added to the pressure on PEPP-PT advocates (Google LLC 2020a). The reason for developing the protocol, as PO#1 stated, was that Apple and Google wanted to enable the Bluetooth background functionality on smartphones, including iPhones, to allow DCT apps to work effectively. While some of the developers on both sides shared different philosophies, pragmatism was applied because both companies wanted to quickly contribute to the containment of the pandemic, leading to a swift agreement (PO#1). The protocol overlaps in functionality with the DP-3T, as Google and Apple were inspired by the protocol (Etherington and Lomas 2020). Likewise, the GAEN relies on a decentralized BLE approach to proximity tracing. However, the two platform owners decided on coring the protocol, i.e., they pushed it from the app layer to the operating system layer (Hoepman 2021). Hence Apple implemented the GAEN on iOS and Google introduced it on Android within Google Play Services (Leith and Farrell 2021). Besides resolving the restrictions of iOS (i.e., BLE), it also ensures proper communication between the operating systems Android and iOS (Google LLC 2020a, c). Google and Apple provide corresponding APIs allowing health authorities to integrate the protocol into their DCT apps. The provision of the GAEN protocol was simultaneously accompanied by a new set of rules (Google LLC 2020b) designed to govern its use based on input and process control measures. For one, the use of the APIs remains reserved for decentralized apps. Additionally, Apple and Google state that no location data can be obtained when using the APIs. Also, only apps that are operated in cooperation with public health authorities are granted access. Eligibility is further “limited to one app per country unless the country has a regional approach” (Google LLC 2020f). Google and Apple additionally announced the so-called Exposure Notification Express, which directly integrates DCT functionalities into the platform core of iOS and Android and therefore does not rely on an app (Apple Inc. 2020c). While the GAEN protocol finally resolved the restrictions of the innovation platform iOS, the tightly regulated access proved to be disappointing for governments advocating centralized approaches (Abboud et al. 2020). Apple and Google justified the exclusion of centralized DCT apps with the fear of the GAEN being repurposed as a surveillance tool:

*After all, it was not the problem with the German solution that Apple had, but Apple said that the release for a centralized approach would then also fundamentally enable abusive apps, which [they] do not expect from Germany, but which other states would then possibly pick up on and then actually develop a surveillance tool out of it. (G#2)*

The group behind the DP-3T project, which strongly criticized centralized protocols, naturally welcomed the decentralized architecture of the GAEN protocol (Doffman 2020b). A few days after the announcement of the GAEN protocol, 300 international scientists in the field of data privacy and protection criticized centralized approaches for contact tracing apps in an open letter. The scientists emphasized that “it is crucial that citizens trust the applications in order to produce sufficient uptake to make a difference in tackling the crisis” (Kaafar et al. 2020, p. 1). This letter was again referred to by several renowned German digital associations, including the Chaos Computer Club, in their own open letter to the German Minister of Health. The letter questioned the centralized approach of the PEPP-PT initiative because of threats to user privacy. Furthermore, the signatories of the letter expressed that “a corona tracing app should, if at all, only be built and programmed on the basis of a decentralized approach—such as the DP-3T […] concept” (D64 et al. 2020, p. 1). The European Commission likewise encouraged health authorities and research institutions to consider a decentralized approach in line with the principle of data minimization (Publications Office of the European Union 2020). Despite the criticism, France, England, Germany, and TraceCOV continued to back centralized approaches. At the time, Germany found itself lacking a possible way to assess the effectiveness of decentralized approaches:

*[…] in the beginning, we lacked the imagination of how to develop such a tracing app, but at the same time evaluate its effectiveness at some point when you don't have any data on how many people are warned by the app and how many of them test positive later. (G#2)*

*On the part of the Ministry of Health, the centralized approach was certainly promoted to our working group, and it was explained why the centralized approach was the more important and better one. Which were also quite conclusive arguments. (G#1)*

Eventually, France publicly demanded Apple and Google adjust their privacy policy as it would block the development of its DCT app (Fouquet 2020). The French government thereby sought to eliminate the technical constraints of centralized apps (Hern 2020) and argued that its centralized solution at the time did not violate European data protection laws (Lomas 2020b). The latter was later confirmed by the National Commission on Informatics and Liberty (CNIL 2020; Morrow 2020). The French junior minister for digital affairs argued that sovereign states must be independent in deciding how to address the challenges caused by the pandemic (Abboud et al. 2020). However, the request, in particular to Apple, to undertake changes to its policy was not reciprocated (Newton 2020a). Eventually, the German Ministry of Health announced on April 26, 2020, a restart of its app development with a decentralized architecture based on the GAEN protocol (Bundesgesundheitsministerium 2020). Germany thus joined Switzerland and Austria, which were among the first adopters of the GAEN (Busvine 2020b). G#1 stated that this change of mind was surprising even within parliamentary circles:

*We ourselves probably never really understood why this switch came so abruptly. That was certainly also influenced by the fact that these companies, namely Google and Apple, were actually pursuing this decentralized approach with their […] protocol. (G#1)*

As stated by G#2, Germany realized that without collaborating with Google and Apple, there would be significant technological barriers that could not be quickly resolved:

*[…] if you didn’t want to use an interface that was provided by Google and Apple, […] you would then encounter significant technological challenges that could not be easily solved without the support of these two players. (G#2)*

G#2 added that the adoption of the GAEN was deemed to be a double-edged sword. However, following the French example and engaging in discussions with the two platform owners was not considered promising:

*It makes you dependent on the big players, but you also notice very quickly that they have solved technological challenges with the API that we hadn’t solved before, and that’s why it’s such a balancing act. (G#2)*

*We really didn't have any other alternative, and pressure would not have been a promising option, because it worked for some decisions in the past, but for this fundamental decision, it was relatively clear that Apple would not budge. (G#2)*

However, G#2 added, that the change of mind was additionally driven by the fact that the previously missing imagination regarding how to obtain data in decentralized approaches such as the GAEN was now available through the use of voluntary data donations and user surveys:

*And now, through this survey and through the data donation, we have really been able to extract very extensive insights into what the app actually does. (G#2)*

Given that centralized apps have suffered considerable reputational damage (Scott et al. 2020), TraceCOV was bound to follow the German government’s shift to a decentralized approach. The consortium recognized that the trust of users in the app itself and its respective operator is decisive in driving adoption. The main drawback for TraceCOV involved the strict input control of both platform owners regarding the GAEN protocol:

*[...] Apple has a rule that only one app per country is allowed in, and of course, that is always the government app, the official one. That is understandable to a certain extent because they want to increase the adoption and do not want to promote a variety of apps. On the other hand, it is, of course, difficult for us. (D#1.1)*

As stated by PO#1, the restriction to one app per country was introduced because Apple did not want to open the Bluetooth background function to every complementor due to security reasons and the potential for improper use. An exception is only made if a country follows a regional approach. However, in Germany, SAP and Deutsche Telekom hold the exclusive right to use the GAEN protocol. In response to the fact that the GAEN APIs cannot be accessed, TraceCOV operates its own contact tracing system developed by CrowdCo based on the DP-3T protocol. Concerning Bluetooth restrictions, the consortium continued to rely on the internally developed ‘wake-up’ workaround, benefiting from the corporate environment in which the app is applied. TraceCOV’s developers expected a sufficiently high coverage by smartphones in the corporate context to mitigate the limitations of iOS devices through the workaround:

*[...] In scenarios where you have Android devices and iPhones and relatively high coverage, it actually works quite well. It is pretty much as close as you can get to the perfect solution from Apple and Google [...]. (D#3.2)*

However, D#3.2 further added that the workaround would not work at low adoption rates as iOS devices would then run the risk of being isolated from other smartphones for a prolonged period. In this case, iPhones would stop performing contact tracing in the background. England, which has introduced a similar ‘wake-up’ method (Vincent 2020), faced that problem (Francisco 2020). While advocating the centralized approach (Newton 2020a), England’s National Health Service (NHS) kept the door open for a shift to the decentralized model of Google and Apple (Newton 2020b). After assessing both the GAEN protocol and the centralized in-house solution, Health Secretary Matt Hancock announced: “Our app won’t work because Apple won’t change that system… and their app can’t measure distance well enough to a standard that we are satisfied with” (Kelion 2020c). England was therefore indecisive and suspended its app development for some time:

*UK then discontinued relatively soon and did not develop for a while, and then came back to us when the political decision was made there to develop a new decentralized solution. They were then two to three months behind us. (G#2)*

Germany, on the other hand, embraced the cooperation with both platform owners and sought to improve the GAEN protocol jointly with Google and Apple, for instance regarding distance measurements:

*[…] we then had discussions with Apple and Google, which led to improvements on their side and improvements on our side […]. (G#2)*

England eventually integrated the GAEN protocol despite its dissatisfactory distance calculations. However, England had to face Apple’s corporate policies once again in 2021. England’s NHS sought to update its app to enable users to upload their history of attended events (Meyer 2021). The update was supposed to guide the relaxation of lockdown rules, but Apple and Google blocked the update via their respective app stores (Kelion 2020a). While the issues were resolved later on, Germany also had to face the strict policies regarding the GAEN API:

*[…] Apple was not thrilled that we wanted to do the [venue] check-in. In the beginning, they were very concerned because they were afraid that location data could be used via this check-in feature. […]. But that was also a point why the check-in [feature] took longer [...]. And we had the same thing again with the vaccination certificates. Here, too, our colleagues in the USA were not enthusiastic because they said it was not part of the core functions [...]. (G#2)*

Meanwhile, in Germany, the privately held Luca-App offered users a way to check in at venues and skip the pen-and-paper registration process. A similar scenario with two separate apps for DCT and check-ins occurred, for instance, in Scotland (Meyer 2021). France ended up being the last governmental representative of centralized approaches in Europe. The app TousAntiCovid (formerly StopCovid) was released in June 2020 (Kelion 2020b). It is based on the centralized “ROBust and privacy-presERving proximity Tracing” (ROBERT) protocol, jointly developed by members of the PEPP-PT group, namely INRIA (France) and Fraunhofer (Germany) (Ahmed et al. 2020). While ROBERT involves the local collection and storage of proximity contacts, the COVID-19 risk exposure is performed on a central server (Castelluccia et al. 2020). Three weeks after the app’s release, the download numbers reached 1.9 million, but only 13 notifications were sent in that period (Dillet 2020). The app collected more data than officially announced and initially struggled with very low adoption rates (Rowe et al. 2020) before eventually being successfully repurposed as a tool enabling the documentation of COVID-19 tests and vaccinations (Schultz et al. 2022). The French app is not interoperable with any of the other European apps (European Commission 2021). In contrast to France, the German app managed to reach high user numbers sooner (Rowe et al. 2020) and is furthermore considered to be highly privacy-preserving, as initial studies suggest (e.g., Krehling and Essex 2021). While France failed to provide users with adequate information concerning the privacy and security of their app (Rowe et al. 2020), Germany’s app and its adoption rates seemed to profit from the close exchange with privacy and security experts:

*As far as data security was concerned, for example, the Chaos Computer Club was invited to the expert discussion. They did not find any leaks. That is very rare. (G#1*)

*If you want to be successful at all with an app like this, it has to have maximum trust from all the entities, consumer watchdogs, Chaos Computer Club, and others who have a significant say in civil society. (G#2*)

The Singaporean government failed to overcome the Bluetooth limitations of TraceTogether. Singapore, therefore, decided against using smartphones and opted for wearables (Government of Singapore 2020). Australia, one of the first adopters of BlueTrace, switched to a QR code-based solution (Ilanbey 2021). The blame for the failure of its DCT app was placed on Apple and Google (Barbaschow 2020). Others, like the Netherlands, began already phasing out their GAEN-based DCT apps in 2022 (PO#1).

### Post standards war: prevention of fragmentation and lock-out

As D#5.1 explained, the Corona-Warn-App does not seem to be suitable for TraceCOV’s customers due to its limited feature scope. Like Germany, TraceCo and CrowdCo were able to alleviate the lack of analyzable data concerning decentralized approaches by relying on voluntary data donations of users. Thus, TraceCOV can still provide its customers with relevant analyses. Furthermore, unlike nationwide DCT apps, TraceCOV has the advantage of being able to bypass the Bluetooth issues due to its expected high coverage in the corporate environment. The standard route to deploy TraceCOV would have been via the public Google Play Store and the Apple App Store. However, Google and Apple categorically decline COVID-19 apps on their transaction platforms. Both companies specify the requirement that COVID-19 apps must be published at least in association with public health authorities (Google LLC 2020d; Apple Inc. 2020b). Like in the case of the GAEN APIs, Apple and Google argue on the grounds of protecting user privacy. D#3.1 implied that both companies thereby want to defend the GAEN protocol as the standard for DCT:

*[…] There is quite a bit of their politics involved: “We actually only want to bring in our interface.” So, there they set up very, very big hurdles. It is not even a competition on the market because nobody from TraceCo says that the German Corona-Warn-App is garbage. They just say: “we want to make it accompanying to it to increase the bandwidth.” (D#3.1)*

As D#3.1 pointed out, TraceCOV is meant to be complementary to the Corona-Warn-App. It is not intended to be a competing product for the official German app. The project sponsor added:

*[…] quite on the contrary because that would harm us at the end of the day and our whole business with the government. (D#5.1)*

Nevertheless, Google and Apple blocked TraceCOV on their respective transaction platforms based on the ‘bouncer’s right.’ PO#1 emphasized that Google and Apple’s intention in providing the GAEN protocol was to assist public health authorities in their fight against the pandemic. Opening the GAEN API to other complementors was never envisioned. D#1.2 referred to this deployment issue the consortium faced as a “potential showstopper.” D#6 added: “If we were a pure start-up, I think I can say openly, we would not have survived this.” The issue is primarily caused by Apple, as Google is less restrictive and offers several alternative distribution options (Google LLC 2020e). Apple, on the other hand, directs providers of COVID-19 apps, which are not approved for the official app store, to use the Apple Developer Program for the deployment to clients (Apple Inc. 2020a). However, for the Apple Developer Program to be viable, both customer and provider must hold an Apple Developer Account (Apple Inc. 2020a). In this vein, one of TraceCOV’s pilot customers has been stuck in the review process for an Apple Developer Account for 3 months at the time of the interview. Besides the route via an Apple Developer Account, the consortium opted for the use of third-party mobile app distribution platforms to deploy their solution to the iOS devices of customers. While both approaches provide a potential way to deploy TraceCOV, they did not offer a long-term solution. The project sponsor commented on the use of third-party mobile app distribution platforms:

*It simply costs too much. And it is terribly complicated. I mean, if every company has an enterprise account (i.e., Apple Developer Account), then it is easy. It works immediately. But that is not the standard. Not every company has its own enterprise account, especially not medium-sized companies, and that is where we actually want to go. (D#5.1)*

In order to fulfill Apple’s input requirements, the consortium was constantly looking for a suitable partner in the healthcare sector. However, even after multiple applications with suitable partners, they were all rejected by Apple. The test management feature of TraceCOV was eventually introduced as a stand-alone browser-based solution.

## Discussion

In the following sections, we discuss our case based on the theoretical foundation of this study. To contrast the adoption of the GAEN, we additionally include the French case which has already been extensively analyzed in the study by Rowe et al. (2020).

### Competing roles and the DCT dilemma

The pandemic led to increased efforts to protect *citizens*, *workers*, and *users* by health authorities, employers, and platform owners, respectively. The role of public health authorities during a pandemic is to ensure the well-being of their citizens and to minimize deaths and severe illnesses. Hence, the countries considered in this part of the study (i.e., Germany and France) initially expressed a preference for centralized approaches, expecting evaluations of DCT data to inform their actions (Rowe et al. 2020). However, it should not be ignored that the centralized approach enjoyed an initial advantage, as it represented essentially the only available approach until the DP-3T consortium split off from the PEPP-PT project. The demands on DCT apps set by the governments examined in this part of the study correspond to the needs of TraceCOV’s customers to maintain their business operations in a safe manner. If companies cannot enable their employees to work from home, yet are committed to preserving their business operations, they need to ensure the safety of their workforce. In Germany, for instance, this is regulated in the German Civil Code (§ 618 BGB) which states that employers are obliged to create an environment in which employees are protected against danger to life and health. Platform owners, on the other hand, need to ensure the health and safety of their ecosystems (Ghazawneh and Henfridsson 2010; Eaton et al. 2015). This need to govern their ecosystems was especially prevalent during the pandemic. Consider, for instance, the wave of malicious apps sparked by the onset of COVID-19, designed by hostile developers to illegally capitalize on users’ fears (e.g., Wang et al. 2021). Hence, while the platform owners aimed to contribute to the fight against the pandemic by enabling DCT apps, they simultaneously sought to avoid potential adverse effects on their ecosystems (see Sect. 5.2). The latter is enabled by their proprietary and decentralized privacy by design approach to DCT which only grants reputable providers access to vulnerable platform resources. As such, different demands arise on the DCT technology and ultimately determine which specific approach, decentralized or centralized, is preferred. Taking into account the respective roles of the various stakeholders, we argue that initially each of the examined stakeholders legitimately favored one over the other approach. However, what then emerged is essentially what Selander et al. (2010, p. 10) refer to as value competitions which they describe as “periods [that] are marked by tensions and struggles […].”

Balancing the different demands on DCT apps proved difficult as they are deeply rooted in the technology itself. While the collection and evaluation of DCT data increase the capability of DCT apps to support containing the pandemic (White and van Basshuysen 2021b; Elmokashfi et al. 2021), it simultaneously introduces stronger privacy concerns, which in turn might translate into lower adoption rates (Chan and Saqib 2021). Given the presence of network effects, however, a significant installed user base represents a prerequisite for suppressing virus transmission using smartphone-based DCT (Hinch et al. 2020). On the other hand, rigorous privacy by design approaches might lead to higher adoption rates while exhibiting disadvantages, especially in terms of fewer insights on the effectiveness of DCT (Riemer et al. 2020; White and van Basshuysen 2021a). As illustrated in Table 5, due to this dualism between evaluations of DCT data to contain the pandemic and minimizing the collection of sensitive data to preserve user privacy (Wareham et al. 2014), contradictory tensions arise (Cameron 1986). The tensions inherent in the technology thus create a dilemma between both competing demands, namely data and privacy (Selander et al. 2010; Smith and Lewis 2011). We argue that this causes a standardization dilemma as the tensions cannot be resolved without at least partially neglecting individual interests when agreeing on a particular standard. While a dualism in the sense of the more data, the less adoption is present (Wareham et al. 2014; Lindgren et al. 2021), voluntary data donations offer a potential resolution strategy by allowing the implementation of a privacy by design approach without entirely compromising on the evaluation of data. This strategy was utilized, for instance, by both the Corona-Warn-App and TraceCOV.

**Table 5:**
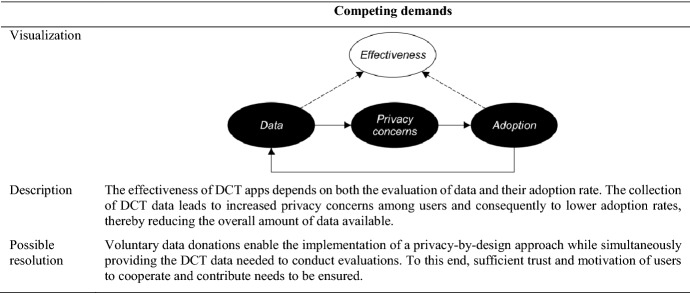
Description of the DCT dilemma. Based on Lindgren et al. (2021) and Wareham et al. (2014)

### The substitution response by Google and Apple

**Fig. 3:**
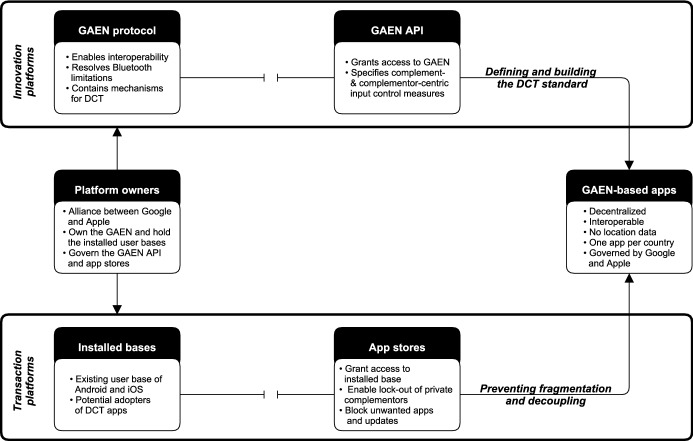
Standardization of digital contact tracing apps based on boundary resources

The alliance consisting of the two platform owners was caught between the desire to quickly contribute to the containment of the pandemic on the one hand, and Apple’s security concerns about opportunistic complementors misusing the Bluetooth background functionality on the other. In addition, as mentioned above, both companies sought to keep malicious apps out of their respective app stores in order to safeguard their installed user base (Google LLC 2020d; Apple Inc. 2020b). Abstention by the platform owners would have rendered DCT infeasible, thus essentially blocking a potentially life-saving crisis measure and risking reputational damage. The imposition of a proprietary standard resolved this tension as it facilitated the development of DCT apps while maintaining control over the respective ecosystems. Such substitution responses by organizations are considered a viable strategy for managing tensions between compliance and non-compliance concerning externally imposed standards (e.g., Okhmatovskiy and David 2012). Therefore, as illustrated in Fig. 3, the platform owners exploited predominantly two distinctive boundary resources to enable the substitution response. The core concepts we refer to in this context are described in Table 6 for the purposes of this study.

**Table 6:** Description of the core concepts

Concept	Description
GAEN-based apps	DCT apps that leverage the technological foundation of the GAEN protocol via the GAEN API, thereby complying with the regulations and specifications jointly defined by the platform owners Google and Apple
GAEN protocol	Technological foundation embedded in the operating system layer of iOS and Android devices that provides the respective smartphones with mechanisms to log users’ encounters and alert them once they have come in close proximity with an infected user
GAEN API	Boundary resource that provides regulated access to the GAEN protocol allowing governmental apps to access the contact tracing data collected in the operating system layer while the platform owners remain in control
Installed user base	Existing iOS and Android users, representing the total number of potential adopters of DCT apps and thus constituting a critical resource for achieving the mass adoption required for DCT to be effective
App store(s)	Boundary resource that provides regulated access to the installed base of Android and iOS allowing app providers to distribute their apps and reach potential adopters while the platform owners can lock out certain complementors and block unwanted complements
Bluetooth limitations	Apple’s predetermined governance measures restricting the Bluetooth functionality for iOS devices due to potential security concerns

First, the GAEN protocol has been made accessible for external complementors via its corresponding API, while at the same time being subject to new rules that allow the platform owners to define the GAEN-based apps according to their own vision (Ghazawneh and Henfridsson 2010). The regulations incorporate both complement- and complementor-centric input control (Croitor and Benlian 2019). The former ensures that apps inherently comply with the platform owners’ policies, such as adhering to mandated privacy standards and refraining from collecting location data. The latter enables the platform owners to govern which complementors are authorized to access the GAEN protocol through the corresponding APIs. Only one provider per country is approved and additionally needs to be associated with a public health authority, thereby excluding private developers, and preventing the misuse of the Bluetooth background functionality. The GAEN API thus serves a twofold role, as it enables access and facilitates generative activities on the one hand but simultaneously restricts access to the GAEN protocol to retrain control on the other (Gawer 2020). When eligible complementors build their app based on the GAEN protocol, process control takes effect as the API mandates a decentralized approach. This process control ultimately results in the fact that apps that are developed based on the GAEN comply with the platform owner’s policies without the need to pre-determine the specific outcomes (Kirsch 1997; Goldbach et al. 2014), for example, in terms of design at the app layer. Although Croitor and Benlian (2019) argue that process control leads to high complexity due to the large number of complementors to be monitored, this is somewhat mitigated in the case of the GAEN protocol due to the “one app per country” rule. Hence, we argue that the platform owners’ substitution response might have been additionally motivated by eliminating redundant apps to facilitate the monitoring of complementors (Wen and Zhu 2019).

Second, compared to other solutions, the GAEN protocol benefits from being technologically superior in an almost artificial manner since it represents the only solution for which the Bluetooth restrictions on iOS devices have been relaxed. This, in turn, leads to an indirect devaluation of alternative solutions (White and van Basshuysen 2021a), as they do not function adequately due to said limitations. However, there are certain apps, such as the one from TraceCo, which are able to bypass the Bluetooth limitations to a large extent due to their unique use case. Such potential standards competitors are effectively excluded from access to the installed user base via the corresponding app stores based on the “bouncer’s right” (Boudreau and Hagiu 2009), specifically by leveraging the centralized input control on the Apple App Store. Besides avoiding the fragmentation of DCT solutions, transaction platforms are used to prevent decoupling (Meyer and Rowan 1977; Westphal and Zajac 2001). Consider the case of England, which eventually adopted the GAEN protocol. When the British government tried to modify its app in a way that would enable location data to be collected, the update was blocked via the respective app stores. In both previously mentioned cases, access to the installed user base was prevented.

Ultimately, our case shows that platform owners can coerce third-party complementors into using boundary resources that they would not have adopted without the powerful influence of the platform owners. Furthermore, leveraging boundary resources allowed the platform owners to combine external design capabilities with their internal innovation capabilities (Selander et al. 2010). Thus, in response to our second research question, we argue that by limiting individual leeway of complementors and subsequently forcing them into the GAEN protocol, Google and Apple were able to ensure the widespread adoption of their proprietary technology. Alternative technologies were devalued by preventing access to valuable platform resources and private developers were locked out based on input control measures (Schilling 1998). This standardization further resulted in the imposition of decentralized DCT as the dominant design since decentralized risk estimation is essentially inscribed into the GAEN protocol by the regulated API (Gallagher 2007). The platform owners Google and Apple thus used their power over the platform resources that enable effective DCT, as well as the transaction platforms that provide access to potential adopters, to coerce complementors into continued subjectification (Hurni et al. 2022). By supplying the technological foundation for DCT apps as a boundary resource, the platform owners enabled DCT for public health authorities while gaining control over DCT and minimizing the risk of adverse consequences for their ecosystems. On the one hand, it can be argued that Google and Apple have quickly resolved the standards war within Europe through their intervention and thus enabled a fast and privacy-preserving digital response to the pandemic (Krehling and Essex 2021). On the other hand, as argued in the introduction of this study, the consequences of such “platform owner-based” (Marhold and Fell 2021, p. 367) standardizations in the context of an international crisis need to be critically examined.

### Trade-offs, tensions, and consequences

**Fig. 4:**
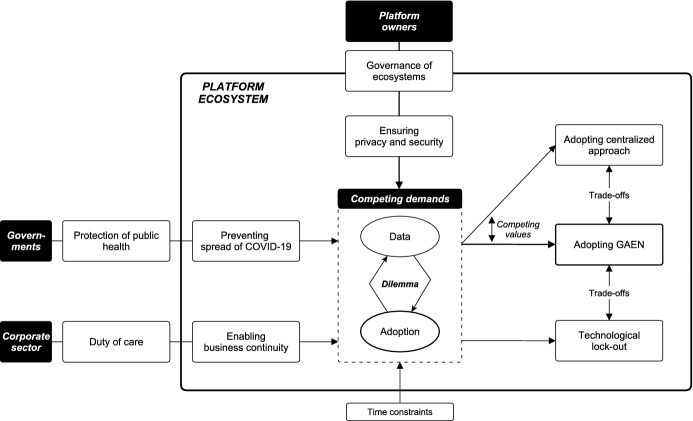
The multi-layered decision situation of DCT. Own illustration based on Selander et al. (2010)

As previously illustrated, the platform owners ultimately used their powerful competitive position within their ecosystems (Selander et al. 2010) to set the GAEN protocol up as the dominant technology while locking out private developers (Schilling 1998). As shown in Fig. 4, we essentially observed two different potential paths from the perspective of the German Ministry of Health. First, the choice to maintain the centralized approach plagued by technological constraints due to the lack of support by Google and Apple or, second, the switch to the technologically superior alternative represented by the emerging standard, namely the GAEN.^2^ In the public debate, decentralized and centralized approaches were ascribed different values for privacy and data evaluation, respectively (White and van Basshuysen 2021b). In this regard, it is worth mentioning that the privacy threats *perceived* by the users are ultimately the crucial factors to consider with respect to end-users’ adoption decisions (Trang et al. 2020). We, therefore, argue that it is of secondary importance to our analysis whether the decentralized approach is in fact more privacy-preserving than its centralized counterpart since the public opinion represents the decisive determinant in terms of adoption. While centralized approaches do not require end-users to voluntarily donate data, they have been publicly criticized by privacy experts (Kaafar et al. 2020; D64 et al. 2020), thus running the risk of low adoption rates (Chan and Saqib 2021). In contrast, health experts argued that the GAEN might be less effective due to the lack of available user data (Albergotti and Harwell 2020), yet privacy experts publicly welcomed the approach taken by Google and Apple (Sharon 2020; Krehling and Essex 2021). If we now consider that the primary drawback of the GAEN protocol can be mitigated to some extent by voluntary data donations, then it seems reasonable to argue that decentralized approaches such as the GAEN balance the paradoxical tensions of the DCT dilemma in a more effective manner. However, the decision is not as simple as it may seem, as we show below.

The switch to the GAEN protocol is accompanied by an increased dependence on and influence by Google and Apple given its proprietary nature (Hanseth and Monteiro 1997; Backhouse et al. 2006). For instance, as our case illustrates, the implementation of updates to incorporate new features such as venue check-ins is subject to the approval of the platform owners and was initially denied. Other apps, like the so-called Luca-App, which was not affected by the special rules governing the GAEN API, were able to implement this function, leading to the Luca-App being the default app for check-ins in Germany until the feature was implemented in the Corona-Warn-App (Bach 2021). Hence, requiring Google’s and Apple’s approval risks delaying or even blocking intended changes to GAEN-based DCT apps that would be considered compliant with the platform owners’ ordinary regulations, thereby limiting the predictability and flexibility of the responsible authorities to tailor their apps to regional COVID-19 developments and policies (Hanseth et al. 1996; Braa et al. 2007). Particularly, public health authorities need to be aware that both platform owners possess the capability to change the modalities of the GAEN. For instance, although PO#1 reported that Google and Apple did not pursue any commercial objective with the GAEN, they can theoretically remove the functionality from their respective operating systems or repurpose it in support of their own needs (Floridi 2020; Boutet et al. 2021; Hoepman 2021). Consider, for example, how mere disagreements between Google and Apple regarding the GAEN could jeopardize the interoperability between Android and iOS DCT apps. Ultimately, the dependence in terms of decisions around governmental DCT apps grants Google and Apple increased influence in public health sectors of sovereign states (Sharon 2020). Hence, when health authorities accept the public–private partnership, the platform owners’ quasi-governmental role becomes essentially legitimized (Leclercq-Vandelannoitte and Aroles 2020; Marhold and Fell 2021). Further, decentralized approaches such as the GAEN protocol are intended to address the risk of potential state surveillance (Fraunhofer AISEC 2020). However, since the code of the GAEN protocol was initially released as closed source, the departure from a centralized approach also entailed a shift in terms of whom end-users would ultimately need to trust (Hoepman 2021). Leith and Farrell (2021, p. 610), for instance, assess the GAEN protocol as highly privacy-preserving, yet argue that it is flawed in that it “leaves users trusting that [Google and Apple] are acting according to their official statements, and are not collecting or storing data.” In the case of the GAEN, then, acceptance of DCT apps depends not only on citizens’ trust in the respective government and the technology itself (Riemer et al. 2020), but also to some extent on the level of trust in the platform owners. Yet, this does not necessarily translate into benefits for users should their government adhere to a centralized solution as exemplified by France.

The centralized French app, for instance, collected more data than initially announced (Rowe et al. 2020). Due to the initial low adoption rates of TousAntiCovid, we suggest that end-users’ trust in centralized approaches had potentially already been damaged by the public debate (White and van Basshuysen 2021a). However, it should not be ignored that although it would have been of crucial importance to build trust with users, especially when using a centralized design, France failed to educate their citizens about the privacy and security of its app (Rowe et al. 2020). In contrast, Germany invited privacy experts (e.g., Chaos Computer Club) to review its app and leveraged an open source approach (Krehling and Essex 2021), potentially contributing to citizens’ trust and acceptance. Adopting what the public perceives as the inferior approach in terms of privacy in favor of public health further carries the risk of being misused as a precedent by future governmental authorities to soften privacy laws under the pretext of crises threats (Urbaczewski and Lee 2020; Rowe 2020). Hence, while the French government made it a point to avoid the dependency on Google and Apple as well as the loss of its digital sovereignty (Abboud et al. 2020; Floridi 2020; Rowe 2020), it did so at the expense of lower adoption rates (Dillet 2020) and the burden of technological limitations (see Sect. 5.2) while risking future health crises being misused for surveillance measures (Urbaczewski and Lee 2020). Furthermore, not following other states in their decision to adopt the GAEN resulted in their app not being interoperable with European solutions, posing an important drawback of remaining with centralized DCT. However, it seems plausible that if Germany, as an influential country (Farrell and Klemperer 2007), had decided against the adoption of the GAEN and thus resided with France, might have altered the course of the standardization process (Wade 1995).

The fact that Apple and Google eventually won the standards war implied for private developers the exclusion from the dominant standard due to regulated APIs (Schilling 1998), resulting in further trade-offs. On the one hand, we argue that the platform owners’ governance policies were instrumental for the timely standardization and the prevention of the fragmentation of the emerging standard. By privileging their own solution within both ecosystems and excluding other approaches and participants, a patchwork of non-interoperable European solutions has been largely avoided. Moreover, the protection and privacy of users was ensured. Potentially harmful apps were blocked from the app stores, and hostile developers who might choose to abuse the Bluetooth background functionality were preemptively hindered by the categorical exclusion of private developers from the GAEN API. Essentially, the platform owners eliminated any market motive to capitalize on the pandemic. On the other hand, we argue that potential adverse consequences resulting from the platform constraints imposed by Google and Apple were introduced. As argued by Trang et al. (2020), end-users have different preferences regarding the specifications of DCT apps. In this vein, the strictly limited access to the GAEN API prevented diverse end-user needs from being captured by multiple interoperable apps. So far, however, we do not know whether multiple interoperable apps would lead to higher or even lower adoption rates. Further, since, according to TraceCOV’s customers, the feature scope of national apps such as the Corona-Warn-App is not sufficient to effectively control COVID-19 in corporate ecosystems, their needs were neglected. In addition, in the long run, there are no incentives for entrepreneurs and innovators to further advance the GAEN or alternative approaches considering the strict input control they would face (Wen and Zhu 2019). Especially when considering that since the release of the GAEN, Apple and Google show little interest in developing their protocol further and if so, as our data shows, it needs to be driven by the public health authorities themselves (Boutet et al. 2021). However, only eligible health authorities can make such contributions, as private developers and entrepreneurs are excluded from the technology and cannot gain experience therewith or share knowledge in order to improve the technology (Arthur 1989; Zhang et al. 2020).

### Conclusion

In conclusion, we argue that adopters of the GAEN prioritized short-term benefits, such as facilitating effective DCT and the rapid diffusion of the corresponding apps, over their digital sovereignty by granting private companies access to policy decisions. Further, the benefits of the timely pan-European standardization may legitimize Apple’s and Google’s strict governance measures (Leclercq-Vandelannoitte and Aroles 2020), while potentially hindering the advancement of the technology for future crises. However, considering the extreme time constraints faced by the stakeholders involved, it seems reasonable to quickly decide on the most viable solution, namely the GAEN protocol, as a comprehensive technological search for the optimal technology to satisfy all needs and avoid long-term pitfalls proved to be largely infeasible. Hence, from a purely technological perspective, we might agree with Liebowitz and Margolis (1999, p. 235) in so far that “good [technologies] win,” especially if there is no time to find the optimal technology.

## Lessons learned from DCT in Europe

**Fig. 5:**
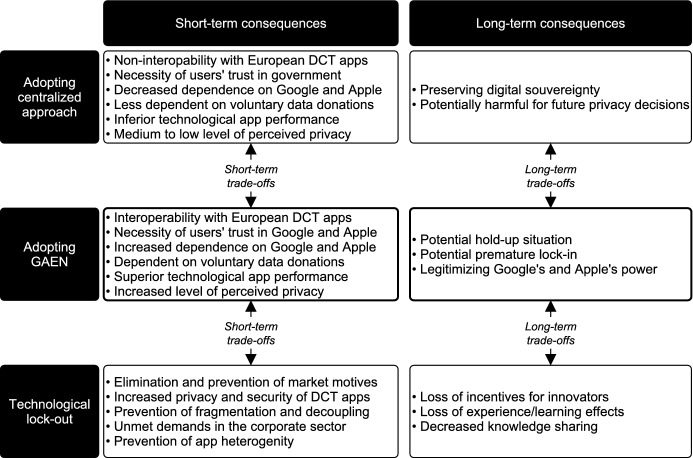
Short-term and long-term trade-offs in the standardization of DCT

We have shown that DCT led to various competing values and goals (Goh and Arenas 2020), but at the same time health crises demand quick decisions from governments. Specifically, the case produced two major interdependent goal conflicts. First, the competing demands on the DCT technology, i.e., the paradoxical tension between ensuring end-user adoption and enabling data evaluations. Second, the competing values regarding the choice of the technological approach to DCT, whereby the adoption of either approach is associated with different short-term and long-term trade-offs (see Fig. 5). The inherent hazard that responsible actors face in such situations, characterized by competing goals and values, lies in the fact that attempting to balance the trade-offs simultaneously may ultimately lead to a state of analysis paralysis (Thacher and Rein 2004). Consider England, for instance, which initially took a two-pronged approach by simultaneously pursuing their own centralized approach as well as the decentralized GAEN, resulting in a temporary halt of their development activities.

With respect to similar multi-layered decision situations, we, therefore, propose a sequential consideration of the different goals and values, thereby obeying the underlying notion of the cycling approach formulated by Thacher and Rein (2004). Cycling offers a rational way to mitigate competing values by temporarily focusing on one goal while shifting awareness to the opposing goal at a later stage. However, it is critical that responsible actors assess which goal to pursue first (Weiner 1998; Thacher and Rein 2004). We, thus, suggest that actors caught in such situations first need to decide which of the competing demands on the technology should be temporarily prioritized, so that the approach most suitable for this purpose may then be chosen, followed by a subsequent effort to address the opposing goals of both decisions. Hence, in contrast to the bias strategy for mitigating value conflicts, whereby individual goals are deliberately ignored to enable decision-making (Stewart 2006; de Graaf et al. 2016), the cycling approach is characterized by the fact that the previously neglected goals are given sufficient attention at a later stage (Thacher and Rein 2004).

In the case of DCT, however, the question remains as to which of the competing demands, i.e., adoption or data, should be given temporal priority. Answering this question requires consideration of whether either option could prevent the opposing goal from being addressed at a later stage (Weiner 1998; Thacher and Rein 2004). In our case, as indicated by the French app,^3^ we argue that prioritizing data may preclude the feasibility of achieving high adoption rates afterward, as users’ trust may have already been damaged. Conversely, as exemplified by Germany, initially focusing on driving the acceptance and diffusion of DCT apps preserves the option of introducing solutions to obtain data at a later stage, as the installed user base can then be exploited through voluntary data donations and user surveys, making the diffusion of DCT apps the initially preferred goal. Since we have established the diffusion of the technology as the primary goal for the time being, the question of which of the two approaches, i.e., GAEN or centralized, is fundamentally superior shifts to which approach is more effective in terms of achieving diffusion. Having this in mind, it seems reasonable to opt for the adoption of the GAEN despite the associated negative consequences (see Fig. 5), as the technology had the potential for substantial adoption by end-users due to its privacy-friendly nature and by app providers (i.e., European health authorities) due to its technological superiority (van de Kaa et al. 2011). After having chosen the GAEN in our chain of reasoning, we can now explore ways to achieve the goal of obtaining data within the limits of this approach. As mentioned earlier, voluntary data donations, for instance, permit data collection despite the decentralized data storage. In this context, the sequential consideration of the conflicting goals allows for innovative ideas that might not have been considered when attempting to balance the conflicting goals simultaneously (Thacher and Rein 2004).

Finally, as already mentioned, in the spirit of the cycling approach, prioritizing the short-term benefits of the GAEN for the sake of diffusion requires addressing the opposing values, i.e., the decreased incentives for innovators, digital sovereignty, and the lock-in threat, later on. However, considering that post-pandemic interest in technologies developed solely for the purpose of mitigating adverse effects of a crisis will greatly decrease, the risk lies in neglecting this last step. Following this line of thought, and assuming that during a crisis, short-term goals take precedence over long-term ambitions (Rowe 2020), we propose that technological decisions should be assigned a legally binding expiration date, which then requires the re-evaluation of choices made under time constraints. Thus, we can ensure that the opposing goals are adequately addressed and not dismissed once the crisis is resolved, allowing for enhanced preparedness for similar situations. As a result, in the case of DCT, policy makers should then be obliged to consider questions of whether the GAEN was effective, how we define the accountability and decision rights in the partnership with the platform owners (Leclercq-Vandelannoitte and Aroles 2020), and what options exist to incentivize innovators in order to advance the technology further.

**Fig. 6:**
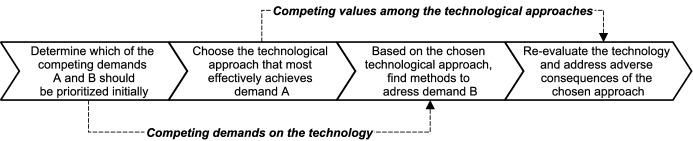
Cycling approach for the introduction of new technologies under time constraints and network effects. Own illustration based on Thacher and Rein (2004)

To conclude, we suggest, that the sequential consideration of the cycling approach might be suitable for policy makers to navigate crisis situations by shifting the question of which goal is more important in general to the question of which goal is temporarily more valuable without neglecting the opposing goals (Thacher and Rein 2004). It should be noted, however, that we have followed the cycling approach solely in its fundamental idea in order to theorize a potential solution for managing such multi-layered decision situations. For instance, Thacher and Rein (2004) anticipate a constant shift back and forth between goals, while we assume a processual progression as illustrated in the exemplary model shown in Fig. 6. In this example, we assume that goal A is prioritized (e.g., diffusion).

## Theoretical and practical implications

First, like other studies on DCT (e.g., Rowe et al. 2020), the primary purpose of our research lies in understanding and explaining. In this sense, we show how various tensions and struggles arise when a tracing information system is introduced in response to a pandemic. We argue that these tensions were driven by the competing demands placed upon the technology itself characterized by the dualism between the need for data to control the disease and the need for data minimization to enable voluntary adoption by end-users. Further, we demonstrate how those competing demands were reinforced by the competing roles of the involved stakeholders, whose actions are interdependent due to the presence of network effects and the resulting need for a technological standard (Weitzel and König 2006). We inform policy makers by inductively identifying various short- and long-term consequences and trade-offs associated with various decision paths. To this end, we expect our findings to support policy makers in future technology decisions under time constraints. With this in mind, we contribute to the preparedness for future public health crises by drawing on the existing literature to illustrate effective strategies for enabling policy makers to engage in goal-directed and rapid decision-making despite the presence of time constraints. Specifically, we apply the underlying notion of the cycling approach proposed by Thacher and Rein (2004) to develop a high-level process model to guide health authorities in navigating technology decisions in future crises. In this context, we propose a built-in expiration date that allows for the re-evaluation of the technology itself as well as the surrounding regulations and partnerships, thereby preventing lock-in scenarios without risking analysis paralyses during the crisis itself.

Second, our results show that a de facto duopoly, such as the one held by Google and Apple, can be considered a viable facilitator in enabling rapid digital crisis responses. On the one hand, the already standardized mobile operating systems Android and iOS offer developers a wide range of options to extend the functional scope of smartphones to address the resulting challenges of crises. In this respect, platform owners play a significant role by enabling and facilitating the reprogrammability (Yoo et al. 2010) of their proprietary technologies by sharing boundary resources and thus granting third-party developers access to standardized platform resources. On the other hand, in the case of complements exhibiting network effects, platform owners can use their coercion power within their ecosystem (Hurni et al. 2022) to rapidly mandate a particular technology as the technological standard, thereby resolving standards wars and leveling the way for a fast digital crisis response. In this vein, Hurni et al. (2022) recently found the prevailing “coaxing only” assumption in platform ecosystems to be incomplete (Parker and Van Alstyne 2018). Our results support their findings by showing that platform owners can coerce third-party complementors into using boundary resources that they might not have adopted without the powerful influence of the platform owners. Thus, in line with the findings of Marhold and Fell (2021), we support the notion of platform owners adopting a quasi-governmental role within their ecosystem. We add to this stream of literature by demonstrating that both innovation and transaction platforms jointly support this coercion power. Both platforms can be used by owners to constrain the individual leeway of complementors in such a way that they can be effectively channeled into compliance.

Third, we add to the recent stream of literature on standards within platform ecosystems (Hein et al. 2019; Tessmann and Elbert 2022) by borrowing the concept of substitution standards from the corporate governance literature (Okhmatovskiy and David 2012) and introducing it as a viable platform strategy. As shown by our case, such substitution responses may be performed by enabling the functions of an externally imposed standard through proprietary boundary resources and coring those features directly into the operating system. In a way, the substitution response is similar to what Hein et al. (2019) call integration through abstraction, referring to situations where complementors develop apps on B2B platforms that are overly specific, prompting the platform owner to aggregate these apps and offer them as boundary resources to the entire platform. While integration through abstraction results from the desire of complementors to monetize their internally developed apps on the platform, the substitution response observed in our case was caused by external developers demanding enhanced access to platform resources. This strategy enabled the controlled opening of the platforms as the new boundary resources simultaneously allowed the introduction of regulation-based securing mechanisms (i.e., process and input control measures). Thus, the substitution response resembles what Ghazawneh and Henfridsson (2013) call diversity resourcing. However, it differs in that the primary purpose is not to stimulate the diversity of third-party complements, but rather to achieve the standardization and homogenization of an emerging app category by controlling the technological architecture of these novel platform extensions. Hence, controlling the technological architecture of complements allows platform owners to end value competitions within their ecosystem without fully undermining the generative efforts of developers (Selander et al. 2010).

## Limitations and opportunities for future research

Our study is not without limitations. First, our study is based on a single case in a unique context. Hence, when attempting to transfer our findings, the context of the study needs to be taken into account. While the rather extreme circumstances may limit the statistical generalizability of our results, they might apply to future crises. Second, the chosen research design for our case study is of an interpretive nature. Although we have consistently strived to present the content of the interviews as truthfully and accurately as possible, we are aware of the potential for biased interpretations. However, we have attempted to counteract such distortions through the deliberate inclusion of secondary data and additional interviews. Third, while we analyzed the case predominantly in light of the standards and platform literature, other theoretical streams might provide further insights or yield alternative explanations for the case under investigation in this study. Fourth, a security and privacy analysis in relation to centralized and decentralized approaches is beyond the scope of this study. Therefore, we would like to direct readers to Krehling and Essex (2021). Fifth, since we focus on Europe, we only address the voluntary adoption of DCT apps. Mandatory use of such apps might lead to other conclusions and interpretations. Finally, we do not consider DCT from an ethical perspective. Hence, we direct readers to Rowe (2020) for a critical review. Further, we invite future researchers to build on our findings and limitations. First, in terms of DCT, future research should examine if differences in terms of adoption exist between a single app per country and multiple interoperable apps from different providers. The latter situation was observed in India (Urbaczewski and Lee 2020). Second, one of the main challenges in standardizing DCT apps arises from the fact that decentralized and centralized approaches are not interoperable. A case in the public sector where similar issues exist is the Online Access Act (Onlinezugangsgesetz) in Germany, which addresses the digitization of administrative services via online portals. The case presents an exciting opportunity for future research and might allow for a contribution to the field of data standards. In addition to data standards, we also see the need for more research on IT standards. Particularly exciting, for instance, is the question of how long it takes for a diffusion process to lose flexibility and for a lock-in effect to occur. In summary, the case analyzed in this study offers several opportunities for future researchers to build upon.

## Appendix

See Table 7.

**Table 7:** Extended data structure with exemplary evidence

Aggregate dimensions	2nd-order themes	1st-order codes	Exemplary evidence
Paradoxical tensions in DCT technology	DCT requires data	Centralized apps increase the effectiveness of DCT	*"The advantage of storing data centrally is that you can do much more with it in terms of analysis" (D#3.1)*
		More data to assess effectiveness through centralized apps	*"[…] there are arguments to follow the centralized approach because then you have a minimum set of data that allows you to evaluate what you are doing" (G#2)*
		Decentralized apps lead to lower data availability	*"[…] it would have been totally exciting […] to do investigations, make analyses, identify hotspots, and so on and so forth [by using a centralized app]" (D#2)*
	DCT requires adoption	Centralized approaches produce more privacy concerns	*"[…] It is to be feared that the low data protection of a centralized approach […] will lead to the erosion of trust in the use of such an app" *(D64 et al. 2020)
		Privacy concerns affect adoption negatively	*"If you want to be successful at all with an app like this, it has to have maximum trust from all the entities […] who have a significant say in civil society" (G#2)*
		Decentralized approaches are more privacy-friendly	*"*Per se*, decentralized is far superior to centralized for privacy reasons […]" (D#3.1)*
Competing roles of stakeholders	Protection of public health	Governments want to maintain public healthcare	*"[…] infection dynamics must remain moderate enough to allow our healthcare system to provide […] treatment to everyone […]" *(Bundespresseamt 2020b)
		Governments need to take swift action	*"That is why we must do everything we can to secure the successes of the last few weeks" *(Bundespresseamt 2020a^a^)
	Governance of ecosystem	Platforms are threatened by a wave of malicious apps	*"We observe that the COVID-19 themed apps as well as malicious ones began to flourish almost as soon as the pandemic broke out worldwide" *(Wang et al. 2021)
		Platform owners want to ensure control over devices	*"Apple [feared] […] other states would […] then actually develop a surveillance tool out of it" (G#2)*
		Platform owners need to ensure health of ecosystems	*"We’re evaluating apps critically to ensure data sources are reputable and that developers presenting these apps are from recognized entities […]" *(*Apple *Inc. 2020b)
	Duty of care	Organizations strive to maintain business operations	*"And of course, it is exciting for all of [our cus tomers]: 'How can I keep my business running?'" (D#6)*
		Large numbers of employees have to work on site	*"We can just send […] our employees to the home office […]. And such companies can't do that. Mechanical engineering can't do that, the automotive industry can't do that, the chemical industry can't do that" (D#4)*
		Organizations are obliged to ensure the safety of workers	*"[…] to ensure […] the matter of self-protection, personal protection, that stems from the duty of care" (D#2)*
Competing demands on DCT	Preventing spread of COVID-19	Governments aim to replace manual tracing with DCT	*"And relatively early on, one thought about how we could use digital means to fight the pandemic efficiently […] with the means of the twenty-first century" (G#1)*
		Governments seek to prevent lockdowns through DCT	*"Countries around the world are developing COVID-19 smartphone apps to limit the spread of coronavirus and relax lockdown restrictions" *(Criddle and Kelion 2020)
		Governments need to assess the effectiveness of DCT	*"[…] people always said, 'we actually need the data, we want as much data as possible to be able to evaluate things […]'" (G#1)*
	Ensuring privacy and security	Platform owners want to enable DCT for governments	*The motivation to develop the GAEN and its APIs was to enable effective contact tracing and thereby support governments. (PO#1)*
		Platform owners intend to prevent the misuse of DCT	*"Apple said that the approval of a centralized approach would […] enable abusive apps […]" (G#2)*
		Non-regulated access to GAEN threatens security of devices	*The restriction "one app per country" was introduced due to security reasons. (PO#1)*
	Enabling business continuity	Organizations seek to leverage DCT to minimize downtimes	*"We want to ensure the issue of business continuity for the companies […] (M#2)*
		Organizations require DCT to coordinate rapid testing	*"So we've talked to big companies who have all their employees tested once a week. […]. And time is a very, very important factor here." (D#6)*
		DCT in organizations requires a broad feature scope	*"However, [companies] also see that ultimately the range of features is not at all sufficient to ultimately handle the corporate needs" (D#5.1)*
Competing values regarding GAEN	Increased app performance	GAEN is superior in battery efficiency	*[…] if you didn’t want to use an interface […] provided by Google and Apple, […] you would then encounter significant technological challenges […]. Specifically, the battery life […]" (G#2)*
		GAEN is superior in distance measurement	*“[…] if you didn’t want to use an interface […] provided by Google and Apple, […] you would then encounter significant technological challenges […]. Specifically, […] the quality of the distance measurement." (G#2)*
		GAEN exhibits a high level of privacy	*"[W]hen Apple and Google launched their contact tracing API […] privacy experts applauded this initiative for its privacy-preserving technical specifications." *(Sharon 2020)
	Decreased digital sovereignty	GAEN is bound to strict specifications	*"Google and Apple […] established strict guidelines to ensure that privacy is safeguarded" *(Google LLC 2020c)
		Dependency on Google and Apple may cause delays	*"[…] Apple was not thrilled that we wanted to do the [venue] check-in. […]. But that was also a point why the check-in [feature] took longer […]" (G#2)*
		Governments and citizens need to trust Google and Apple	*"This leaves users trusting that [Google and Apple] are acting according to their official statements, and are not collecting or storing data" *(Krehling and Essex 2021)
Platform constraints affecting the development	Reduced customization	Adoption of GAEN restricts the feature scope	*"Here, too, our colleagues in the USA were not enthusiastic because they said it was not part of the core functions […]" (G#2)*
		Adoption of GAEN complicates regional adjustments	*"[…] Google and Apple have been blocking England and Wales’s National Health Service (NHS) from rolling out an update to its contact-tracing app" *(Meyer 2021)
	Reduced app variety	Google and Apple discourage multiple interoperable apps	*"[…] Apple has a rule that only one app per country is allowed in. […] On the other hand, it is, of course, difficult for us" (D#1.1)*
		Google and Apple prevent alternative approaches	*"The result is that there is exactly this one COVID-19 app in Germany. And this is also the only one in the store that can be downloaded." (D#3.1)*
	Thwarted innovation	Adoption of GAEN inhibits incentives for entrepreneurs	*"[The] […] adoption of the GAEN solution […] stopped the continuous process of improvement of digital contact tracing" *(Boutet et al. 2021)
		Google and Apple hamper learning for entrepreneurs	*"Anyone wishing to compare specification to the code cannot do so without Google and Apple’s invitation" *(Krehling and Essex 2021)
Time constraints affecting the development	Need for viable solution	Pandemic requires a quick solution	*Pragmatism was displayed because both companies wanted to quickly contribute to the containment of the pandemic. (PO#1)*
		Preferring ineffective over privacy-invasive solutions	*“[W]e’d rather the thing doesn’t work so well than have a problem at the data protection level” (G#1)*
	Search for optimal technology	Comprehensive technological search requires time	*The platform owners quickly created a global solution, naturally skipping certain steps that would have been taken under normal conditions when setting up such a collaborative project. (PO#1)*
		No benchmarks or precedents for DCT were available	*"So it's really hard to assess. There is *de facto* no precedent." (D#1.2)*

^a^The sources refer to the reference list provided in the main article.

## Footnotes

1 The members of the consortium and the application itself are replaced by pseudonyms.

2 Another path would be the adoption of the DP-3T protocol, as exemplified by Switzerland. However, Google and Apple remain in control in this scenario, hence we do not consider this option separately. For more details we kindly direct readers to Vaudenay (2020b).

3 It should be noted that the French app recorded very high adoption rates later since it was repurposed into a tool for proving vaccination certificates, while the contact tracing function was disregarded by users (Schultz et al. 2022).
